# Association of Radiotherapy for Rectal Cancer and Second Gynecological Malignant Neoplasms

**DOI:** 10.1001/jamanetworkopen.2020.31661

**Published:** 2021-01-08

**Authors:** Xu Guan, Ran Wei, Runkun Yang, Zhao Lu, Enrui Liu, Zhixun Zhao, Haipeng Chen, Ming Yang, Zheng Liu, Zheng Jiang, Xishan Wang

**Affiliations:** 1 National Cancer Center/Cancer Hospital, Chinese Academy of Medical Sciences & Peking Union Medical College, Beijing, China; 2 The Second Affiliated Hospital of Harbin Medical University, Harbin, China

## Abstract

**Importance:**

Radiotherapy is a common treatment for rectal cancer, yet the risk of second gynecological malignant neoplasms (SGMNs) in patients with rectal cancer undergoing radiotherapy have not been adequately studied.

**Objective:**

To investigate the association between radiotherapy and the risk of individual types of SGMN in patients with rectal cancer and assess survival outcomes.

**Design, Setting, and Participants:**

A large population-based cohort study was designed to identify the risk of SGMNs in patients with rectal cancer diagnosed from January 1973 to December 2015. The statistical analysis was conducted from September 2019 to April 2020. The study was based on the 9 cancer registries of Surveillance, Epidemiology, and End Results database. A total of 20 142 female patients with rectal cancer in localized and regional stage were included.

**Exposure:**

Receipt of neoadjuvant radiotherapy for rectal cancer.

**Main Outcomes and Measures:**

The development of an SGMN defined as any type of GMN occurring more than 5 years after the diagnosis of rectal cancer. The cumulative incidence of SGMNs was estimated by Fine-Gray competing risk regression. Poisson regression was used to evaluate the radiotherapy-associated risk for SGMNs in patients undergoing radiotherapy vs patients not undergoing radiotherapy. The Kaplan-Meier method was used to assess the survival outcomes of patients with SGMNs.

**Results:**

Of 20 142 patients, 16 802 patients (83.4%) were White and the median age was 65 years (interquartile range, 54-74 years). A total of 5310 (34.3%) patients were treated with surgery and radiotherapy, and 14 832 (65.7%) patients were treated with surgery alone. The cumulative incidence of SGMNs during 30 years of follow-up was 4.53% among patients who received radiotherapy and 1.53% among patients who did not. In competing risk regression analysis, undergoing radiotherapy was associated with a higher risk of developing cancer of the uterine corpus (adjusted hazard ratio, 3.06; 95% CI, 2.14-4.37; *P* < .001) and ovarian cancer (adjusted hazard ratio, 2.08; 95% CI, 1.22-3.56; *P* = .007) compared with those who did not receive radiotherapy. The dynamic radiotherapy-associated risks (RR) for cancer of the uterine corpus significantly increased with increasing age at rectal cancer diagnosis (aged 20-49 years: adjusted RR, 0.79; 95% CI, 0.35-1.79; *P* = .57; aged 50-69 years: adjusted RR, 3.74; 95% CI, 2.63-5.32; *P* < .001; aged ≥70 years: adjusted RR, 5.13; 95% CI, 2.64-9.97; *P* < .001) and decreased with increasing latency since rectal cancer diagnosis (60-119 months: adjusted RR, 3.22; 95% CI, 2.12-4.87; *P* < .001; 120-239 months: adjusted RR, 2.72; 95% CI, 1.75-4.24; *P* < .001; 240-360 months: adjusted RR, 1.95; 95% CI, 0.67-5.66; *P* = .22), but the dynamic RR for ovarian cancer increased with increasing latency since rectal cancer diagnosis (60-119 months: adjusted RR, 0.70; 95% CI, 0.26-1.89; *P* = .48; 120-239 months: adjusted RR, 2.26; 95% CI, 1.09-4.70; *P* = .03; 240-360 months: adjusted RR, 11.84; 95% CI, 2.18-64.33; *P* = .004). The 10-year overall survival among patients with radiotherapy-associated cancer of the uterine corpus was significantly lower than that among matched patients with primary cancer of the uterine corpus (21.5% vs 33.6%; *P* = .01).

**Conclusions and Relevance:**

Radiotherapy for rectal cancer was associated with an increased risk of cancer of the uterine corpus and ovarian cancer. Special attention should be paid to reduce radiotherapy-associated SGMNs and improve their prognosis.

## Key Points

**Question:**

Is radiotherapy for rectal cancer associated with the increasing risk of second gynecological malignant neoplasms and prognosis in women with rectal cancer?

**Findings:**

In this cohort study of 20 142 patients with rectal cancer, radiotherapy for rectal cancer was associated with an increased risk of second cancer of the uterine corpus and ovarian cancer. Development of cancer of the uterine corpus was associated with a worse prognosis.

**Meaning:**

Results of this study suggest that special attention is warranted to reduce the risk of second gynecological malignant neoplasms in the treatment and follow-up of patients with rectal cancer after radiotherapy.

## Teaser

This cohort study assesses the association between radiotherapy and the risk of individual types of second gynecological malignant neoplasms in patients with rectal cancer and estimates survival outcomes for patients with secondary cancers.

## Introduction

During the last decades, several large randomized trials have demonstrated that neoadjuvant radiotherapy plays a well-established role in the improvement of locoregional control of rectal cancer but is not associated with increased long-term survival.^1,2,3^ Therefore, the benefits and adverse events of radiotherapy should be carefully balanced when considering all potential effects of radiotherapy. Radiotherapy-associated second primary cancer (SPC) is a rare but notable late complication of cancer therapy.^4^

Studies assessing the risk of SPC are inconsistent regarding the role of radiotherapy for rectal cancer treatment.^5,6,7^ Although some studies have demonstrated an increased risk of overall SPCs after radiotherapy for rectal cancer treatment, others have reported no obvious increased risk.^8,9,10^ Because of the likelihood of pelvic organs receiving a greater radiotherapy dose than organs in nonpelvic areas, it is necessary to understand the risk of SPCs in organs within the irradiated volume.^9,11,12^ Assessments of the potential risk of radiotherapy-associated SPC for pelvic cancers, including malignant neoplasms of prostate and bladder, have verified the idea of an increased risk of SPCs in patients undergoing pelvic radiotherapy.^13,14,15^ However, whether the risk of second gynecological malignant neoplasms (SGMNs) is increased after radiotherapy for rectal cancer has not been well addressed.

Although previous studies^8,9,10,16,17,18,19,20^ have investigated the risk of SGMNs among patients with rectal cancer treated with radiotherapy, the current research on this topic is limited and contradictory. Most studies oversimplified the risk estimation by grouping various types of gynecological malignant neoplasms (GMNs) into 1 broad category, without consideration of the great tumor heterogeneity and differences in pathogenesis among SGMN entities.^8,9,10,16,17,18,19,20^ The understanding of radiotherapy-associated SGMN has prognostic and therapeutic implications but has been rarely studied. Therefore, we performed this study with the aims of investigating radiotherapy and the risk of individual types of SGMN and assessing patient survival outcomes.

## Methods

### Database and Participants

Female patients diagnosed with rectal cancer as the first primary cancer were identified from 9 registries of the Surveillance, Epidemiology and End Results (SEER) database between January 1, 1973, and December 31, 2015. All primary cancer sites were coded according to the *International Classification of Diseases for Oncology, Third Edition*. The patients included were pathologically diagnosed with rectal cancer (C19.9) or rectosigmoid cancer (C20.9). The tumor stage was restricted to the localized and regional stage, and the details of tumor stage are shown in eTable 1 in the Supplement. The exclusion criteria included patients in whom rectal cancer were not their first primary cancer, patients who were aged younger than 20 years, patients with distant stage, patients who survived less than 5 years after rectal cancer diagnosis, patients who did not undergo surgery, and patients with missing data on radiotherapy, surgery, age, tumor stage, race, survival status or follow-up information. This study has been approved by the Ethics Committee of Cancer Hospital, Chinese Academy of Medical Sciences. The access to and use of SEER data did not require informed patient consent. This study followed the Strengthening the Reporting of Observational Studies in Epidemiology (STROBE) reporting guideline for cohort studies.

### Treatment Interventions for Rectal Cancer

Patients with rectal cancer were classified into 2 groups according to initial treatment modality. The radiotherapy group was composed of patients with rectal cancer who received surgery and neoadjuvant external-beam radiotherapy, and the no-radiotherapy group was composed of patients who received surgery alone. We excluded patients who received other types of radiotherapy (ie, brachytherapy, combination therapy). The SEER database collected data only on the initial course of cancer treatment, and the radiotherapy doses were not registered in the SEER database.

### Definition and Follow-up of SGMNs

The primary outcome of this study was the development of an SGMN, which was defined as any type of GMN occurring more than 5 years after the treatment of rectal cancer because of at least a 5-year latency period from radiotherapy exposure to solid tumor occurrence.^21^ The SEER program adheres to the *International Classification of Diseases for Oncology, Third Edition* guidelines to distinguish SPCs from recurrent disease. To obtain comprehensive estimations for the risk of SGMNs, we first estimated the risk for all types of combined SGMNs and then separately estimated the risk in different types of female genital organs, including the cervix uteri (C53.0-C53.9), corpus and uterus (C54.0-C54.9, C55.9), ovary (C56.9) and other female genital organs as 1 group (C51.0-C51.9, C52.9, C57.0-C57.9, C58.9). The follow-up for SGMNs began 5 years after rectal cancer diagnosis and ended at the date of diagnosis of any SGMN, all-cause death, or after 30 years of follow-up, whichever occurred first. The cut-off point for follow-up was defined as January 1, 2016 (April 2019 SEER data release).

### Statistical Analysis

Fine-Gray competing risk regression analysis was used to assess the cumulative incidence of SGMN development. Experiencing a non-SGMN and dying of all causes were considered competing events by calculating hazard ratios (HRs) and 95% CIs for SGMN occurrence. The multivariable risk model was built by using a backward selection procedure with variables with 2-sided *P* < .05 in univariable analyses, which was considered statistically significant and included in multivariable analyses. χ^2^ tests were used to compare categorical data, or Fisher exact test when frequencies were below 5. Mann-Whitney test were used for analysis of continuous variables with a normal and nonnormal distribution, respectively. This procedure was performed with R software, version 3.5.3 (R Project for Statistical Computing).

The radiotherapy-associated risk (RR) was estimated by using Poisson regression analysis with the relative risk and 95% CIs of SGMN development for patients with rectal cancer who received radiotherapy compared with those who did not receive radiotherapy, and the RRs were calculated with R software version 3.5.3. Then, Poisson regression analysis was used to calculate the standardized incidence ratio (SIR) and 95% CIs. The SIR was defined as the ratio of observed incidence SGMNs among rectal cancer survivors to the incidence of GMNs in the US general population. The SIRs were calculated with SEER*Stat 8.3.6. Both RRs and SIRs were adjusted for the age at rectal cancer diagnosis and the calendar year of rectal cancer diagnosis in our analysis. The details of statistical methods are shown in eFigure 2 in the Supplement. To further evaluate the dynamic risks and incidence for SGMNs associated with radiotherapy, we calculated the RRs and SIRs stratified by latency time since rectal cancer diagnosis, age at rectal cancer diagnosis, and year of rectal cancer diagnosis.

To evaluate the prognosis of SGMNs, the Kaplan-Meier method was used to calculate the 10-year overall survival for SGMNs and only primary GMN, and *P* values were calculated with the log-rank test. Overall survival was defined as the time from SGMN diagnosis to death from any cause. The only primary GMN was defined as the patients diagnosed with only GMN and without any other cancers throughout their lifetime. Propensity score matching was used to reduce possible bias for survival comparison. These analyses were performed using R software (version 3.5.3).

## Results

### Patient Characteristics

A total of 22 589 patients with rectal cancer were identified (eFigure 1 in the Supplement). After excluding the patients with nonmatching data, 20 142 patients remained in final cohort, 16 802 patients (83.4%) were White, and the median age was 65 years (interquartile range, 54-74 years). The median follow-up time was 140 months (interquartile range, 92-212 months). A total of 14 832 patients (65.7%) were in the no-radiotherapy group and 5310 patients (34.3%) were in the radiotherapy group. The baseline characteristics of patients with rectal cancer by treatment modality are shown in Table 1. After a latency of 5 years, 176 patients (1.2%) in the no-radiotherapy group and 144 patients (2.7%) in the radiotherapy group developed SGMN. The baseline characteristics of patients who developed SGMN are shown in eTable 2 in the Supplement.

**Table 1.** Comparisons of Baseline Characteristics of Patients With Rectal Cancer by Treatment Modality

Characteristic	No. (%)		*P* value
	Radiotherapy (n = 5310)	No radiotherapy (n = 14 832)	
Age at rectal cancer diagnosis, median (IQR), y	61 (52-70)	66 (56-75)	<.001^a^
Age at rectal cancer diagnosis, y			
20-49	1047 (19.7)	1767 (12)	<.001^b^
50-69	2886 (54.4)	7230 (48.7)	
≥70	1377 (25.9)	5835 (39.3)	
Year of rectal cancer diagnosis, median (IQR)	1999 (1991-2006)	1993 (1984-2002)	<.001^a^
Year of rectal cancer diagnosis			
1975-1984	503 (9.5)	4095 (27.6)	<.001^b^
1985-1994	1228 (23.1)	4063 (27.4)	
1995-2004	1984 (37.4)	3983 (26.9)	
≥2005	1595 (30)	2691 (18.1)	
Race			
White	4449 (83.8)	12 353 (83.3)	<.001^b^
Black	352 (6.6)	1170 (7.9)	
Other^c^	509 (9.6)	1309 (8.8)	
Tumor grade			
Grade I/II	3992 (75.2)	9899 (66.7)	<.001^b^
Grade III/IV	777 (14.6)	1141 (7.7)	
Unknown	541 (10.2)	3792 (25.6)	
Tumor stage			<.001^b^
Localized	1742 (32.8)	11 393 (76.8)	
Regional	3568 (67.2)	3439 (23.2)	
Tumor histology			
Adenocarcinoma	4831 (91)	14 181 (95.6)	<.001^b^
Mucous tumor	360 (6.8)	490 (3.3)	
Other	119 (2.2)	161 (1.1)	
Tumor size, cm			
<2	191 (3.6)	827 (5.6)	<.001^b^
≥2	1306 (24.6)	1323 (8.9)	
Unknown	3813 (71.8)	12 682 (85.5)	
Chemotherapy			
No	1293 (24.4)	13 892 (93.7)	<.001^b^
Yes	4017 (75.6)	940 (6.3)	
Follow-up time of patients with rectal cancer, mo, median (IQR)	120.5 (93-175)	154 (99-232)	<.001^a^
Total person-years at risk	65 397	205 487	
Latency between rectal cancer and SGMN, mo, median (IQR)	110 (69.3-156.5)	123.5 (63.3-137.8)	<.001^a^
Patients who developed SGMN	144 (2.7)	176 (1.2)	<.001^b^

Abbreviations: IQR, interquartile range; SGMN, second gynecological malignant neoplasm.

^a^ *P* values were calculated using the Mann-Whitney test for continuous variables and χ^2^ test.

^b^ *P* values were calculated using the Mann-Whitney test for categorical variables.

^c^ Other includes American Indian/Alaska Native and Asian/Pacific Islander.

### Cumulative Incidences of SGMNs

**Figure 1.**
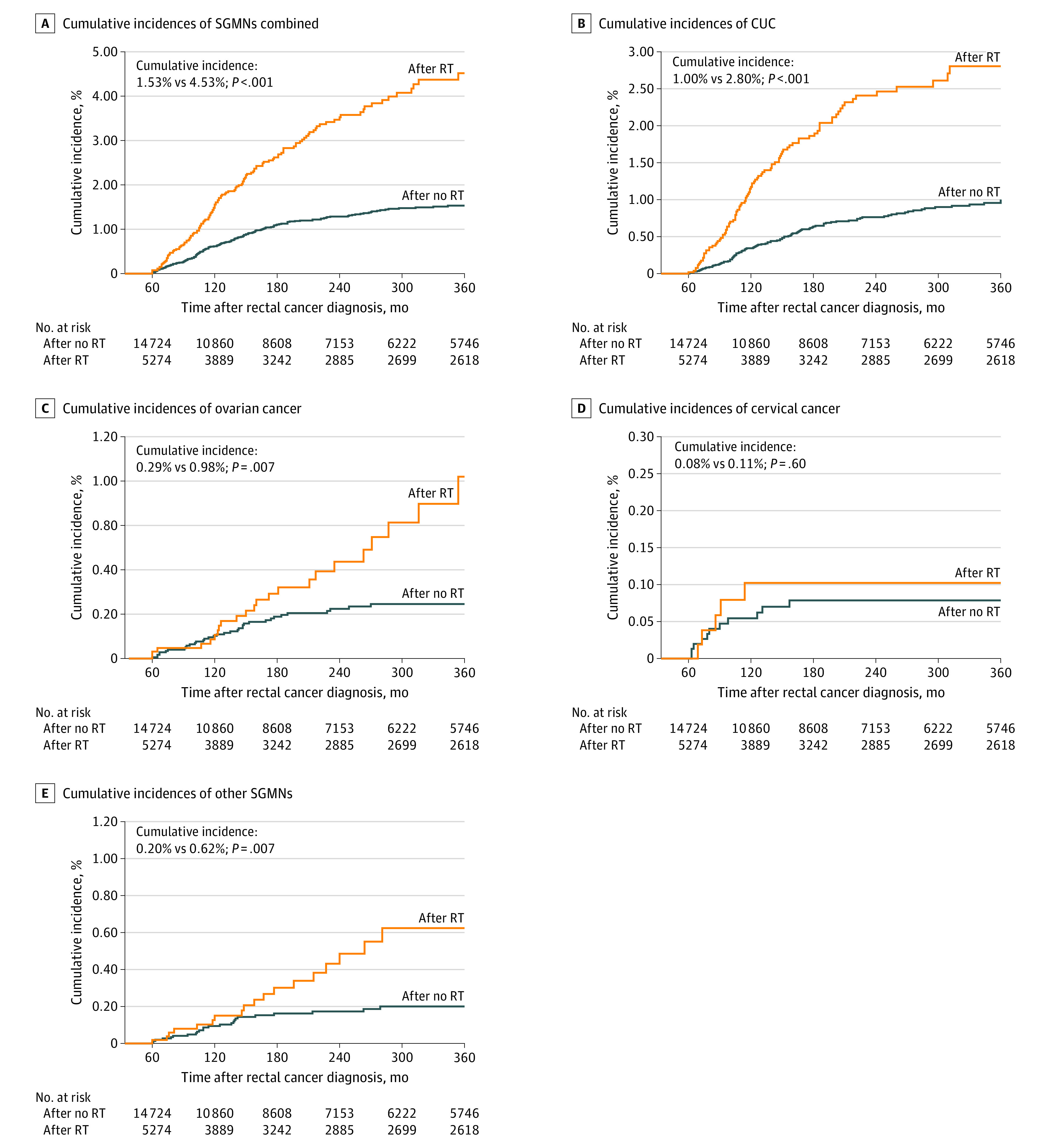
Comparisons of Cumulative Incidence of Second Gynecological Malignant Neoplasma (SGMNs) Between Patients Who Received Radiotherapy (RT) and Patients Who Did Not Receive RT *P* values were calculated with the Fine-Gray test. CUC indicates cancer of the uterine corpus.

The cumulative incidence of combined SGMNs was 2.16% after rectal cancer diagnosis; the incidences were 1.53% in patients with no radiotherapy and 4.53% in patients receiving radiotherapy (*P* < .001) (Figure 1A). In organ-specific analyses, the cumulative incidences of cancer of the uterine corpus, ovarian cancer, and other SGMNs were significantly higher in the radiotherapy group than the no-radiotherapy group, including cancer of the uterine corpus (1.00% vs 2.80%; *P* < .001), ovarian cancer (0.29% vs 0.98%; *P* = .007), and other SGMNs (0.20% vs 0.62%; *P* = .007), but no difference was observed for cervical cancer (Figure 1B-E).

### Risk of SGMNs Attributable to Radiotherapy

All variables listed in Table 1 were selected for univariable competing risk regression analysis (eTables 3-7 in the Supplement). In multivariable analysis, radiotherapy was associated with a higher risk of developing combined SGMNs (adjusted HR, 2.99; 95% CI, 2.23-4.02; *P* < .001). In analyses of each type of SGMN, increased risks were observed in cancer of the uterine corpus (adjusted HR, 3.06; 95% CI, 2.14-4.37; *P* < .001) and ovarian cancer (adjusted HR, 2.08; 95% CI, 1.22-3.56; *P* = .007) but not in cervical cancer or other SGMNs (Table 2). In subgroup analyses, the increased risk of developing cancer of the uterine corpus and ovarian cancer was associated with radiotherapy in most subgroups with HRs greater than 1.00 (eTables 8-12, and eFigures 3 and 4 in the Supplement).

**Table 2.** Risk of Developing SGMNs in Patients With Rectal Cancer by Statistical Method^a^

SGMNs	Multivariable competing risk regression(RT vs NRT)		Poisson regression (RT vs NRT)		Poisson regression (RT vs US general population)		Poisson regression (NRT vs US general population)	
	Adjusted HR (95% CI)	*P* value	Adjusted RR (95% CI)	*P* value	Adjusted SIR (95% CI)	*P* value	Adjusted SIR (95% CI)	*P* value
Combined SGMNs	2.99 (2.23-4.02)	<.001	2.83 (2.24-3.60)	<.001	2.32 (1.95-2.73)	<.05	0.80 (0.69-0.93)	<.05
Cancer of the uterine corpus	3.06 (2.14-4.37)	<.001	3.19 (2.39-4.26)	<.001	2.88 (2.33-3.50)	<.05	0.93 (0.76-1.12)	NS
Ovarian cancer	2.08 (1.22-3.56)	.007	2.26 (1.30-3.91)	.004	1.34 (0.85-2.01)	NS	0.54 (0.38-0.75)	<.05
Cervical cancer	NA	NA	1.44 (0.48-4.30)	.52	1.12 (0.37-2.62)	NS	0.65 (0.33-1.17)	NS
Other SGMNs	2.02 (0.71-5.57)	.180	2.72 (1.43-5.16)	.002	2.79 (1.65-4.41)	<.05	0.98 (0.63-1.46)	NS

Abbreviations: HR, hazard ratio; NRT, no radiotherapy; NA, not applicable; NS, no significance; RC, rectal cancer; RR, radiotherapy-associated risk; RT, radiotherapy; SGMN, second gynecological malignant neoplasm; SIR, standardized incidence ratio.

^a^ Fine-Gray competing risk regression analyses were used to calculate the HRs and 95% CIs for SGMN in patients with rectal cancer treated with RT vs patients not treated with RT. Covariables that were significant in univariable competing risk regression analysis (*P* < .05) are included in the multivariable analysis. The HR (RT vs NRT) for cervical cancer in univariable competing risk regression was not significant (*P* > .05), so the cervical cancer data were left blank in multivariable competing risk regression. Poisson regression analyses were used to calculate the RR and 95% CIs of SGMNs for patients with RT vs patients with NRT. Similarly, Poisson regression analyses were used to calculate the SIR and 95% CIs of SGMNs for patients with RT and NRT vs the US general population. Both RR and SIR were adjusted for age at RC diagnosis and calendar year of RC diagnosis in our analysis.

The RRs were calculated to confirm the risk of SGMNs attributable to radiotherapy. We found that the adjusted RR of additional risk for combined SGMNs was 2.82 (95% CI, 2.24-3.56, *P* < .001), and increased risk was also observed for cancer of the uterine corpus (adjusted RR, 3.19; 95% CI, 2.39-4.26; *P* < .001), ovarian cancer (adjusted RR, 2.26; 95% CI, 1.30-3.91; *P* = .004), and other SGMNs (adjusted RR, 2.72; 95% CI, 1.43-5.16; *P* = .002) (Table 2). We then found that the SIR for combined SGMNs was 2.32 (95% CI, 1.95-2.73, *P* < .05) in patients who received radiotherapy, and the SIRs for patients who underwent radiotherapy were significantly higher for cancer of the uterine corpus (SIR, 2.88; 95% CI, 2.33-3.5; *P* < .05) and other SGMNs (SIR, 2.79; 95% CI, 1.65-4.41; *P* < .05) (Table 2).

### Dynamic Risk and Incidence Evaluation for SGMNs

**Figure 2.**
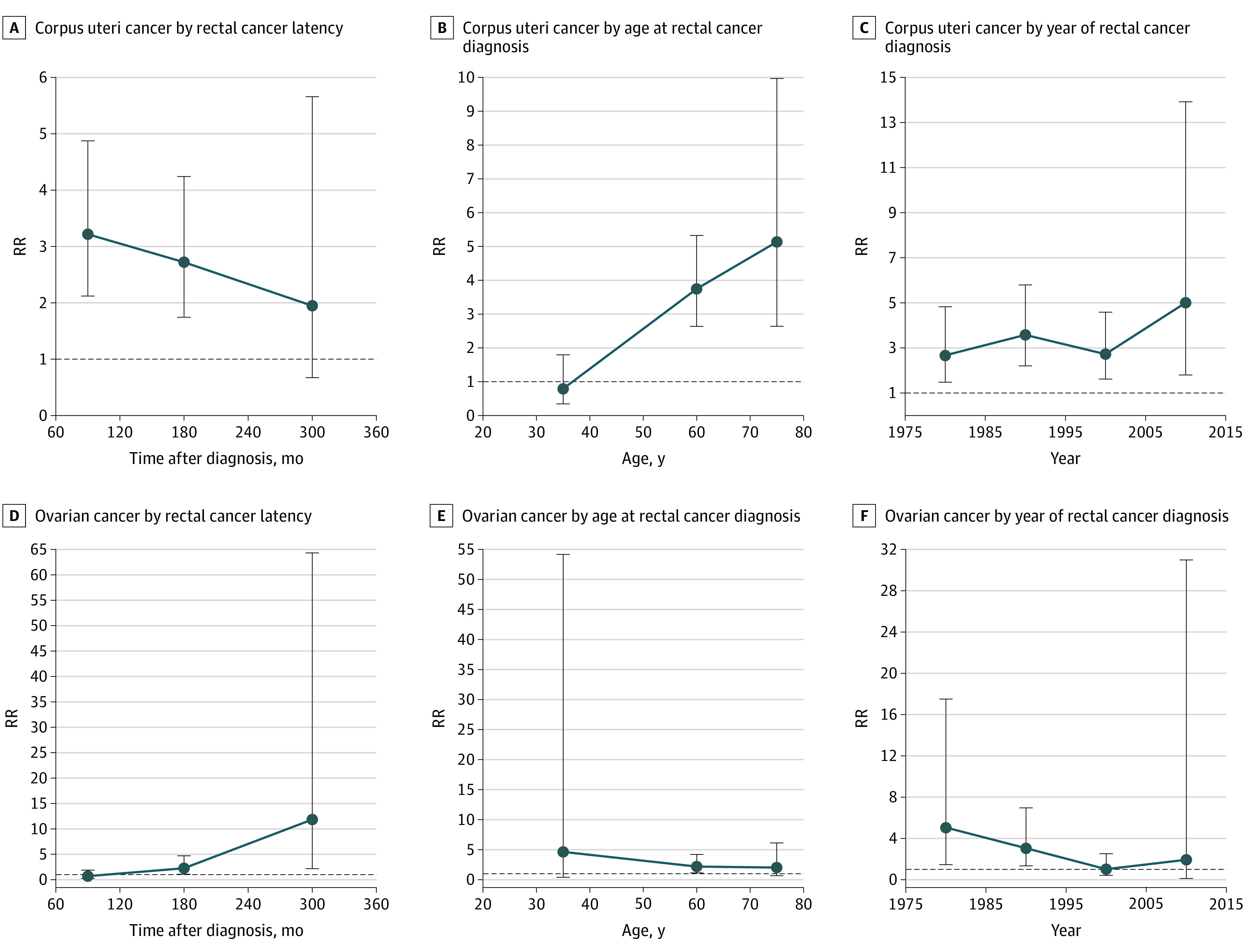
Dynamic Radiotherapy-Associated Risk (RR) Plots Adjusted RRs and 95% CIs of developing cancer of the uterine corpus and ovarian cancer in patients treated with radiotherapy vs patients treated without radiotherapy are plotted.

To assess the risks of SGMNs attributable to radiotherapy, we established 3 dynamic RR plots based on latency period, time of rectal cancer diagnosis, and age at rectal cancer diagnosis (Figure 2; eTables 13-17 in the Supplement). In the dynamic latency-RR plot, an increased risk of cancer of the uterine corpus was observed in the early latency, but this risk presented a downward trend in the late latency (60-119 months: adjusted RR, 3.22; 95% CI, 2.12-4.87; *P* < .001; 120-239 months: adjusted RR, 2.72; 95% CI, 1.75-4.24; *P* < .001; 240-360 months: adjusted RR, 1.95; 95% CI, 0.67-5.66; *P* = .22) (Figure 2A; eTable 14 in the Supplement). In the dynamic age-RR plot, no increased risk of cancer of the uterine corpus was observed in patients aged 20 to 50 years with rectal cancer, but the risk gradually increased and peaked at an age of older than 70 years (20-49 years: adjusted RR, 0.79; 95% CI, 0.35-1.79; *P* = .57; 50-69 years: adjusted RR, 3.74; 95% CI, 2.63-5.32; *P* < .001; ≥70 years: adjusted RR, 5.13; 95% CI, 2.64-9.97; *P* < .001) (Figure 2B; eTable 14 in the Supplement). In the dynamic diagnosis time-RR plot, a slightly increased risk of cancer of the uterine corpus was observed after radiotherapy in the years from 1975 to 1984, and this risk remained elevated up to the years from 2005 to 2015 (1975-1984: adjusted RR, 2.67; 95% CI, 1.48-4.83; *P* = .001; 1985-1994: adjusted RR, 3.58; 95% CI, 2.21-5.79; *P* < .001; 1995-2004: adjusted RR, 2.72; 95% CI, 1.62-4.59; *P* < .001; ≥2005: adjusted RR, 5.00; 95% CI, 1.80-13.92; *P* = .002) (Figure 2C; eTable 14 in the Supplement). However, we observed 3 opposite risk trends in 3 RR plots of ovarian cancer compared with the trends in cancer of the uterine corpus, such as in the latency-RR plot (60-119 months: adjusted RR, 0.70; 95% CI, 0.26-1.89; *P* = .48; 120-239 months: adjusted RR, 2.26; 95% CI, 1.09-4.70; *P* = .03; 240-360 months: adjusted RR, 11.84; 95% CI, 2.18-64.33; *P* = .004; Figure 2D, Supplement Table 15), age-RR plot (20-49 years: adjusted RR, 4.63; 95% CI, 0.40-54.17; *P* = .22; 50-69 years: adjusted RR, 2.19; 95% CI, 1.13-4.21; *P* = .02; ≥70 years: adjusted RR, 2.02; 95% CI, 0.67-6.12; *P* = .21; Figure 2E, Supplement Table 15) and diagnosis time-RR plot (1975-1984: adjusted RR, 5.06; 95% CI, 1.46-17.55; *P* = .01; 1985-1994: adjusted RR, 3.05; 95% CI, 1.34-6.96; *P* = .008; 1995-2004: adjusted RR, 1.02; 95% CI, 0.41-2.52; *P* = .97; ≥2005: adjusted RR, 1.93; 95% CI, 0.12-31.02; *P* = .64) (Figure 2D-F; eTable 14 and eTable 15 in the Supplement).

In addition, we evaluated the dynamic SIRs for patients treated with radiotherapy and patients not treated with radiotherapy. For patients not treated with radiotherapy, no increased incidences of cancer of the uterine corpus (eFigure 5A-C in the Supplement) were observed compared with the incidence in the US general population in 3 plots. For patients who underwent radiotherapy, the SIRs for cancer of the uterine corpus presented similar findings as the RR plots (eFigure 5A-C in the Supplement). In analyses of SIRs for ovarian cancer, we found that SIRs for ovarian cancer in patients who did not receive radiotherapy were significantly lower than those in the US general population in 3 plots (eFigure 5D-F in the Supplement), but no significant increased incidence of ovarian cancer was observed for patients treated with radiotherapy compared with the US general population (eFigure 5D-F in the Supplement). The details of RRs and SIRs were shown in eTables 18-22 in the Supplement.

### Survival Outcome of SGMNs

**Figure 3.**
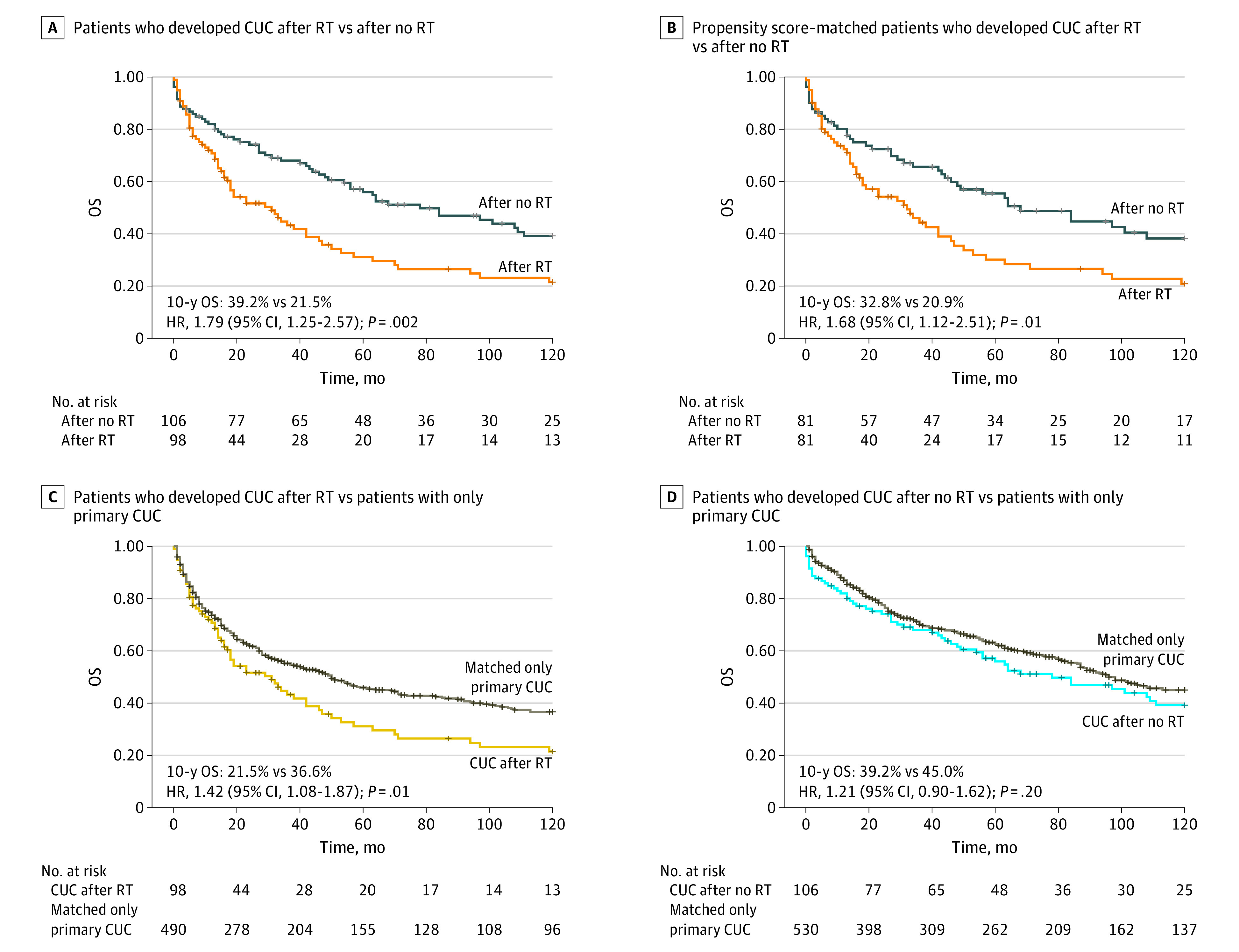
Survival Outcome of Second Gynecological Malignant Neoplasms Hazard ratios (HRs) were calculated using Cox proportional hazards regression models. CUC indicates cancer of the uterine corpus; OS, overall survival; RT,radiotherapy.

We compared survival between patients with cancer of the uterine corpus after radiotherapy and no radiotherapy, and the 10-year overall survival of patients who developed cancer of the uterine corpus after radiotherapy was significantly lower than that of patients after no radiotherapy, both before propensity score matching (Figure 3A) and after propensity score matching (Figure 3B). We then used matched only primary cancer of the uterine corpus patients as a control group by using propensity score matching. We observed that the 10-year overall survival for patients who developed cancer of the uterine corpus after radiotherapy was significantly lower than that for matched patients with only primary cancer of the uterine corpus (10-year overall survival, 21.5% vs 36.6%; *P* = .01) (Figure 3C), and no difference was observed between patients without radiotherapy and matched patients with only primary cancer of the uterine corpus (Figure 3D). Information on only primary GMNs and survival analyses for combined SGMNs, ovarian cancer, cervical cancer and other SGMNs were shown in eTables 23-27, and eFigures 6-9 in the Supplement.

## Discussion

This is, to our knowledge, the first large population-based study to present the risk dynamics and prognosis of individual types of SGMN after radiotherapy among patients with rectal cancer. In our study, the key findings are as follows. First, radiotherapy was considered an important risk factor for the development of cancer of the uterine corpus and ovarian cancer in patients with rectal cancer. Second, the incidence of cancer of the uterine corpus after radiotherapy was higher than that in the US general population. Third, the risks for developing cancer of the uterine corpus after radiotherapy increased with age, increased with diagnosis time, and decreased with latency, but the opposite trends were observed for ovarian cancer. Fourth, cancer of the uterine corpus after radiotherapy presented a poor prognosis.

Previous studies^8,9,10,16^ assessing the risk of SGMNs after radiotherapy treatment for rectal cancer showed conflicting results (eTable 28 in the Supplement); there are several reasons for these variations in results, including the definition of SGMNs, latency period selection, length of follow-up duration, methodology and sample size of the cohort population. Birgisson et al^9^ and Wiltink et al^8^ found that radiotherapy was not associated with an increased risk of developing SGMNs in patients with rectal cancer. However, only 8 and 10 SGMNs were observed in these 2 studies, which lacked statistical power to support their conclusions. Rombouts et al^10^ reported that SGMNs (combined malignant neoplasms of the corpus uteri, cervix uteri, ovary, vulva, and vagina) occurred more frequently in patients with rectal cancer who received radiotherapy, but this study did not perform risk analyses for each type of SGMN. In addition, Kendal et al^17^ found that an increased risk of developing SGMNs, including cancer of the uterine corpus and cervical cancer as 1 group, after radiotherapy treatment, but they did not consider the great heterogeneity among different types of GMNs. To better address this issue, we separately estimated the risk for each type of SGMN in our study.

The issue of latency cut-off point was determined to be associated with the risk of SPC occurrence after radiotherapy, which may be the main reason for the conflicting results regarding the risk of developing ovarian cancer from different studies.^21^ Wang et al^19^ reported that preoperative radiotherapy increased the risk of SPCs in the uterus but not the cervix or ovary after a latency period of more than 1 year. Similarly, Warschkow et al^16^ performed a cohort study to evaluate the risk for SPCs with a 1-year latency after radiotherapy treatment for rectal cancer and found that an increased risk of SGMNs after rectal cancer involved malignant neoplasms of the endometrium only, not the ovary, cervix, or vulva. In our study, we identified SGMNs that occurred after a 5-year latency because the 5-year latency period from radiotherapy exposure to solid tumor occurrence allowed for a more reasonable conclusion to be drawn.^4^ To further confirm the latency influence on risk evaluation, we repeatedly analyzed the risk of SGMN using a 1-year cut-off latency, and found that the risk for cancer of the uterine corpus after radiotherapy continued to be increased, but no risk for other types of SGMNs.

The statistical methods used for analysis are essential for the interpretation of the results. We used both competing risk regression and Poisson regression to assess the risk of SGMNs. Herein, we assessed the increased risks of cancer of the uterine corpus and ovarian cancer after radiotherapy, but other types of SGMNs presented conflicting results according to different statistical method. We compared the incidence of SGMNs in patients who survived rectal cancer to the US general population, which could provide a more comprehensive understanding of the association between radiotherapy and the incidence of SGMNs.

In our analyses, we assessed the dynamic risk and incidence of SGMNs. The highest radiotherapy-associated risk for developing ovarian cancer was found after a latency of more than 20 years, but for cancer of the uterine corpus, the highest risk was within 5 to 10 years. This finding suggests that long-term follow-up may be warranted for the detection of ovarian cancer after radiotherapy, but follow-up for cancer of the uterine corpus should be considered in the early latency. An increased risk of cancer of the uterine corpus was observed with increasing age at diagnosis but was significantly decreased for ovarian cancer. This finding showed that both young patients and patients aged 50 years or older with rectal cancer who underwent radiotherapy faced a high risk of cancer of the uterine corpus. The mechanism underlying these opposite results between cancer of the uterine corpus and ovarian cancer warrants further study. Despite the improvements in radiotherapy treatment for rectal cancer in recent decades associated with better local control, our findings showed that the risk of cancer of the uterine corpus increased to 2.7-fold from 1975 to 1984 and gradually increased to 5.0-fold after 2005. This finding may be associated with changes in radiotherapy dose and radiotherapy modality over time. During the study period from 1973 to 2015, radiotherapy treatment modality has shifted toward hypofractionation, which may underlie the higher incidence of cancer of the uterine corpus in more recent years.

Compared with nonradiotherapy-associated GMNs, the clinical phenotype and prognosis of radiotherapy-associated SGMNs may present great heterogeneity. In our study, we found that patients with radiotherapy-associated cancer of the uterine corpus had worse survival than matched control patients with only primary cancer of the uterine corpus. However, no survival differences between radiotherapy-associated GMNs and only primary GMNs were observed for other types of GMNs. This finding suggests that the occurrence of radiotherapy-associated cancer of the uterine corpus might be due to the induction of other genetic signaling pathways after radiotherapy exposure, which is different from the normal genetic pathway of only primary cancer of the uterine corpus development. Owing to the varied genetic phenotype, radiotherapy-associated cancer of the uterine corpus is resistant to conventional treatment and presents worse prognosis. However, no genomic data in the SEER database could be used to interpret the genetic features of cancer of the uterine corpus after radiotherapy exposure.

### Strengths and Limitations

The strengths of the present study include a long follow-up period to discover potential SGMNs as well as a large observation population with relatively homogenous treatment exposure identified from the SEER database. This study has limitations. First, a lack of randomization of the initial treatment for rectal cancer may be associated with potential biases. However, the occurrence of SGMNs may not only be associated with radiotherapy exposure but may also be affected by other crucial risk factors, such as lifestyle, genetic background, environmental factors, and other cancer-related treatments.^22^ Therefore, it is impossible to balance all factors between the 2 treatment types. Instead, we adjusted all confounding risk factors using a multivariable risk competing model to reduce the potential bias associated with the lack of randomization. Second, we could not determine the associations between modality of radiotherapy, radiotherapy dose, and number of administrations and the risk of SGMNs. Third, the SEER database records only the initial treatment information of rectal cancer, and whether patients with rectal cancer received delayed radiotherapy in subsequent treatment is unknown, which may potentially misclassify the patients in the radiotherapy group into the no-radiotherapy group. However, this limitation is unlikely to affect our main conclusion but reflects only an underestimation of the increased risk attributable to radiotherapy.

## Conclusions

In this cohort study, several methodologies were used together to assess an increased risk of developing cancer of the uterine corpus and ovarian cancer in female patients with rectal cancer who received radiotherapy, and the risk of SGMN was evaluated from several dimensions, which together could provide a meaningful reference for the treatment and follow-up of SGMNs in patients with rectal cancer after radiotherapy.

## Notes

Supplement. **eFigure 1.** Flow Diagram **eFigure 2.** Statistical Methods Annotation **eFigure 3.** Subgroup Analyses of Competing Risk Regression for the Risk of Developing Corpus Uteri Cancer **eFigure 4.** Subgroup Analyses of Competing Risk Regression for the Risk of Developing Ovarian Cancer **eFigure 5.** Dynamic Standardized Incidence Ratios **eFigure 6.** Survival Comparisons Between RC Patients Who Developed SGMs **eFigure 7.** Survival Comparisons Between RC Patients Who Developed OC **eFigure 8.** Survival Comparisons Between RC Patients Who Developed CC **eFigure 9.** Survival Comparisons Between RC Patients Who Developed Other Types of SGMs **eTable 1.** The Description of Tumor Stage of Rectal Cancer in SEER Program **eTable 2.** Comparisons of Baseline Characteristics of RC Patients Who Developed SGMs by Treatment Type **eTable 3.** Univariable and Multivariable Competing Risk Regression Analysis of Risk of Developing Combined SGMs in RC Patients **eTable 4.** Univariable and Multivariable Competing Risk Regression Analysis of Risk of Developing Corpus Uteri Cancer in RC Patients **eTable 5.** Univariable and Multivariable Competing Risk Regression Analysis of Risk of Developing Ovarian Cancer in RC Patients **eTable 6.** Univariable and Multivariable Competing Risk Regression Analysis of Risk of Developing Cervical Cancer in RC Patients **eTable 7.** Univariable and Multivariable Competing Risk Regression Analysis of Risk of Developing Other SGMs in RC Patients **eTable 8.** Subgroup Analyses of Hazard Ratios of Developing Combined SGMs in RC Patients Who Received RT Versus RC Patients Who Did Not Receive RT **eTable 9.** Subgroup Analyses of Hazard Ratios of Developing Corpus Uteri Cancer in RC Patients Who Received RT Versus Those Who Did Not Receive RT **eTable 10.** Subgroup Analyses of Hazard Ratios of Developing Ovarian Cancer in RC Patients Who Received RT Versus Those Who Did Not Receive RT **eTable 11.** Subgroup Analyses of Hazard Ratios of Developing Cervical Cancer in RC Patients Who Received RT Versus Those Who Did Not Receive RT **eTable 12.** Subgroup Analyses of Hazard Ratios of Developing Other SGMs in RC Patients Who Received RT Versus Those Who Did Not Receive RT **eTable 13.** Radiation-attributed Risk of Combined SGMs by Age at RC Diagnosis, Latency and Year of RC Diagnosis **eTable 14.** Radiation-attributed Risk of Corpus Uteri Cancer by Age at RC Diagnosis, Latency and Year of RC Diagnosis **eTable 15.** Radiation-attributed Risk of Ovarian Cancer by Age at RC Diagnosis, Latency and Year of RC Diagnosis **eTable 16.** Radiation-attributed Risk of Cervical Cancer by Age at RC Diagnosis, Latency and Year of RC Diagnosis **eTable 17.** Radiation-attributed Risk of Other SGMs by Age at RC Diagnosis, Latency and Year of RC Diagnosis **eTable 18.** Standardized Incidence Ratio of Combined SGMs by Age at RC Diagnosis, Latency and Year of RC Diagnosis **eTable 19.** Standardized Incidence Ratio of Corpus Uteri Cancer by Age at RC Diagnosis, Latency and Year of RC Diagnosis **eTable 20.** Standardized Incidence Ratio of Ovarian Cancer by Age at RC Diagnosis, Latency and Year of RC Diagnosis **eTable 21.** Standardized Incidence Ratio of Cervical Cancer by Age at RC Diagnosis, Latency and Year of RC Diagnosis **eTable 22.** Standardized Incidence Ratio of Other SGMs by Age at RC Diagnosis, Latency and Year of RC Diagnosis **eTable 23.** Patients Characteristics of Corpus Uteri Cancer and Matched Only Primary Corpus Uteri Cancer **eTable 24.** Patients Characteristics of Ovarian Cancer and Matched Only Primary Ovarian Cancer **eTable 25.** Patients Characteristics of Cervical Cancer and Matched Only Primary Cervical Cancer **eTable 26.** Patients Characteristics of Other SGMs and Matched Other Only Primary SGMs **eTable 27.** Patients Characteristics of Corpus Uteri Cancer Before and After PSM Matching **eTable 28.** Studies of The Risk of Developing SGMs After Radiation Therapy Among RC Patients
