# Supplementary material for: Association of Radiotherapy for Rectal Cancer and Second Gynecological Malignant Neoplasms
Source: JAMA Netw Open. 2021 Jan 8;4(1):e2031661. doi: 10.1001/jamanetworkopen.2020.31661 (PMC7794669; doi:10.1001/jamanetworkopen.2020.31661)
Supplement: Supplement. — eFigure 1. Flow Diagram eFigure 2. Statistical Methods Annotation eFigure 3. Subgroup Analyses of Competing Risk Regression for the Risk of Developing Corpus Uteri Cancer eFigure 4. Subgroup Analyses of Competing Risk Regression for the Risk of Developing Ovarian Cancer eFigure 5. Dynamic Standardized Incidence Ratios eFigure 6. Survival Comparisons Between RC Patients Who Developed SGMs eFigure 7. Survival Comparisons Between RC Patients Who Developed OC eFigure 8. Survival Comparisons Between RC Patients Who Developed CC eFigure 9. Survival Comparisons Between RC Patients Who Developed Other Types of SGMs eTable 1. The Description of Tumor Stage of Rectal Cancer in SEER Program eTable 2. Comparisons of Baseline Characteristics of RC Patients Who Developed SGMs by Treatment Type eTable 3. Univariable and Multivariable Competing Risk Regression Analysis of Risk of Developing Combined SGMs in RC Patients eTable 4. Univariable and Multivariable Competing Risk Regression Analysis of Risk of Developing Corpus Uteri Cancer in RC Patients eTable 5. Univariable and Multivariable Competing Risk Regression Analysis of Risk of Developing Ovarian Cancer in RC Patients eTable 6. Univariable and Multivariable Competing Risk Regression Analysis of Risk of Developing Cervical Cancer in RC Patients eTable 7. Univariable and Multivariable Competing Risk Regression Analysis of Risk of Developing Other SGMs in RC Patients eTable 8. Subgroup Analyses of Hazard Ratios of Developing Combined SGMs in RC Patients Who Received RT Versus RC Patients Who Did Not Receive RT eTable 9. Subgroup Analyses of Hazard Ratios of Developing Corpus Uteri Cancer in RC Patients Who Received RT Versus Those Who Did Not Receive RT eTable 10. Subgroup Analyses of Hazard Ratios of Developing Ovarian Cancer in RC Patients Who Received RT Versus Those Who Did Not Receive RT eTable 11. Subgroup Analyses of Hazard Ratios of Developing Cervical Cancer in RC Patients Who Received RT Versus Those Who Did Not Receive [file jamanetwopen-e2031661-s001.pdf]

Supplemental Online Content

Guan X, Wei R, Yang R, et al. Association of radiotherapy for rectal cancer and second gynecological malignant neoplasms. *JAMA Netw Open*. 2021;4(1):e2031661. doi:10.1001/jamanetworkopen.2020.31661

eFigure 1. Flow Diagram

eFigure 2. Statistical Methods Annotation

eFigure 3. Subgroup Analyses of Competing Risk Regression for the Risk of Developing Corpus Uteri Cancer

eFigure 4. Subgroup Analyses of Competing Risk Regression for the Risk of Developing Ovarian Cancer

eFigure 5. Dynamic Standardized Incidence Ratios

eFigure 6. Survival Comparisons Between RC Patients Who Developed SGMs

eFigure 7. Survival Comparisons Between RC Patients Who Developed OC

eFigure 8. Survival Comparisons Between RC Patients Who Developed CC

eFigure 9. Survival Comparisons Between RC Patients Who Developed Other Types of SGMs

eTable 1. The Description of Tumor Stage of Rectal Cancer in SEER Program

eTable 2. Comparisons of Baseline Characteristics of RC Patients Who Developed SGMs by Treatment Type

eTable 3. Univariable and Multivariable Competing Risk Regression Analysis of Risk of Developing Combined SGMs in RC Patients

eTable 4. Univariable and Multivariable Competing Risk Regression Analysis of Risk of Developing Corpus Uteri Cancer in RC Patients

eTable 5. Univariable and Multivariable Competing Risk Regression Analysis of Risk of Developing Ovarian Cancer in RC Patients

eTable 6. Univariable and Multivariable Competing Risk Regression Analysis of Risk of Developing Cervical Cancer in RC Patients

eTable 7. Univariable and Multivariable Competing Risk Regression Analysis of Risk of Developing Other SGMs in RC Patients

eTable 8. Subgroup Analyses of Hazard Ratios of Developing Combined SGMs in RC Patients Who Received RT Versus RC Patients Who Did Not Receive RT

eTable 9. Subgroup Analyses of Hazard Ratios of Developing Corpus Uteri Cancer in RC Patients Who Received RT Versus Those Who Did Not Receive RT

eTable 10. Subgroup Analyses of Hazard Ratios of Developing Ovarian Cancer in RC Patients Who Received RT Versus Those Who Did Not Receive RT

© 2021 Guan X et al. *JAMA Network Open.*

eTable 11. Subgroup Analyses of Hazard Ratios of Developing Cervical Cancer in RC Patients Who Received RT Versus Those Who Did Not Receive RT

eTable 12. Subgroup Analyses of Hazard Ratios of Developing Other SGMs in RC Patients Who Received RT Versus Those Who Did Not Receive RT

eTable 13. Radiation-attributed Risk of Combined SGMs by Age at RC Diagnosis, Latency and Year of RC Diagnosis

eTable 14. Radiation-attributed Risk of Corpus Uteri Cancer by Age at RC Diagnosis, Latency and Year of RC Diagnosis

eTable 15. Radiation-attributed Risk of Ovarian Cancer by Age at RC Diagnosis, Latency and Year of RC Diagnosis

eTable 16. Radiation-attributed Risk of Cervical Cancer by Age at RC Diagnosis, Latency and Year of RC Diagnosis

eTable 17. Radiation-attributed Risk of Other SGMs by Age at RC Diagnosis, Latency and Year of RC Diagnosis

eTable 18. Standardized Incidence Ratio of Combined SGMs by Age at RC Diagnosis, Latency and Year of RC Diagnosis

eTable 19. Standardized Incidence Ratio of Corpus Uteri Cancer by Age at RC Diagnosis, Latency and Year of RC Diagnosis

eTable 20. Standardized Incidence Ratio of Ovarian Cancer by Age at RC Diagnosis, Latency and Year of RC Diagnosis

eTable 21. Standardized Incidence Ratio of Cervical Cancer by Age at RC Diagnosis, Latency and Year of RC Diagnosis

eTable 22. Standardized Incidence Ratio of Other SGMs by Age at RC Diagnosis, Latency and Year of RC Diagnosis

eTable 23. Patients Characteristics of Corpus Uteri Cancer and Matched Only Primary Corpus Uteri Cancer

eTable 24. Patients Characteristics of Ovarian Cancer and Matched Only Primary Ovarian Cancer

eTable 25. Patients Characteristics of Cervical Cancer and Matched Only Primary Cervical Cancer

eTable 26. Patients Characteristics of Other SGMs and Matched Other Only Primary SGMs

eTable 27. Patients Characteristics of Corpus Uteri Cancer Before and After PSM Matching

eTable 28. Studies of The Risk of Developing SGMs After Radiation Therapy Among RC Patients

This supplemental material has been provided by the authors to give readers additional information about their work.

© 2021 Guan X et al. *JAMA Network Open.*

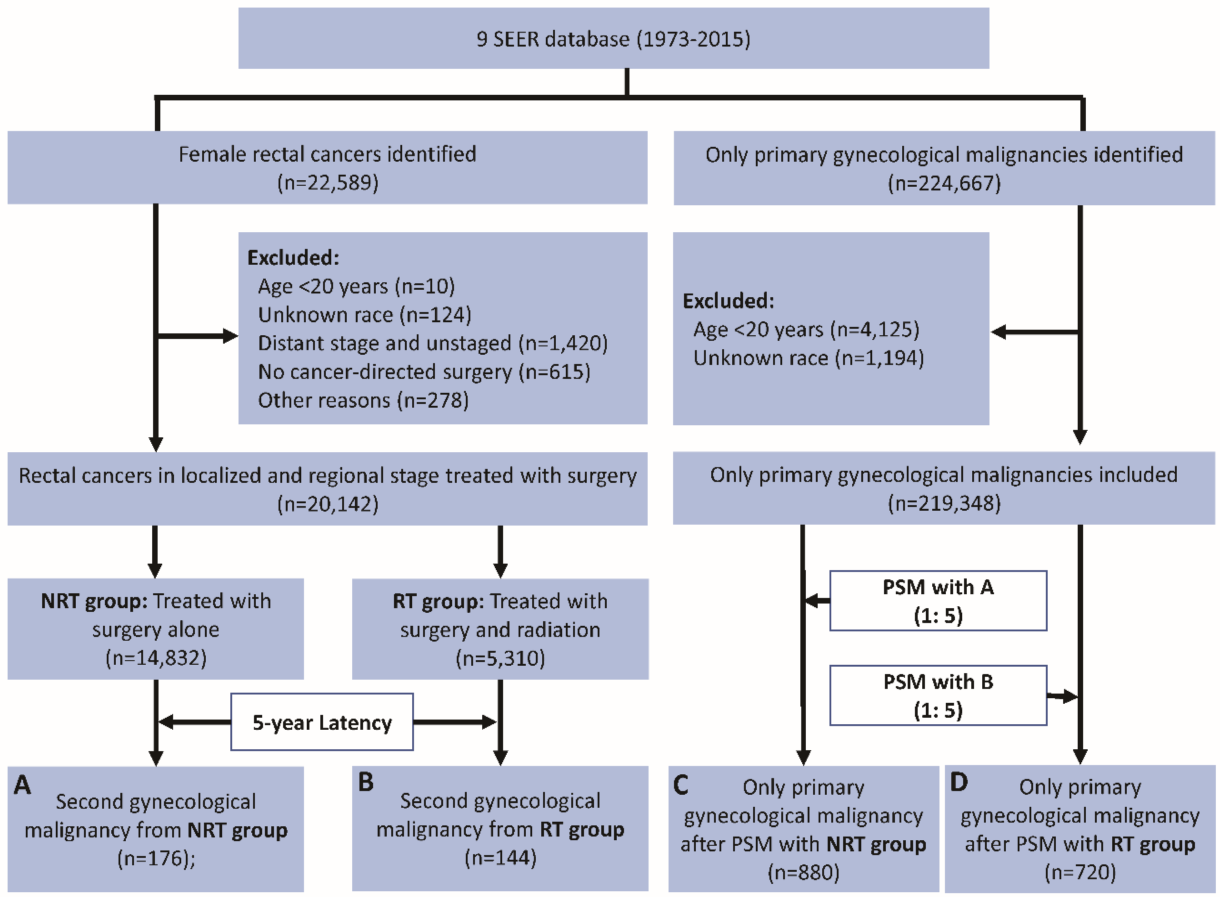

eFigure 1. Flow diagram.

Abbreviations: RT, radiation therapy; NRT, no radiation therapy; SEER, Surveillance, Epidemiology and End Results; PSM, propensity score matching.

© 2021 Guan X et al. *JAMA Network Open.*

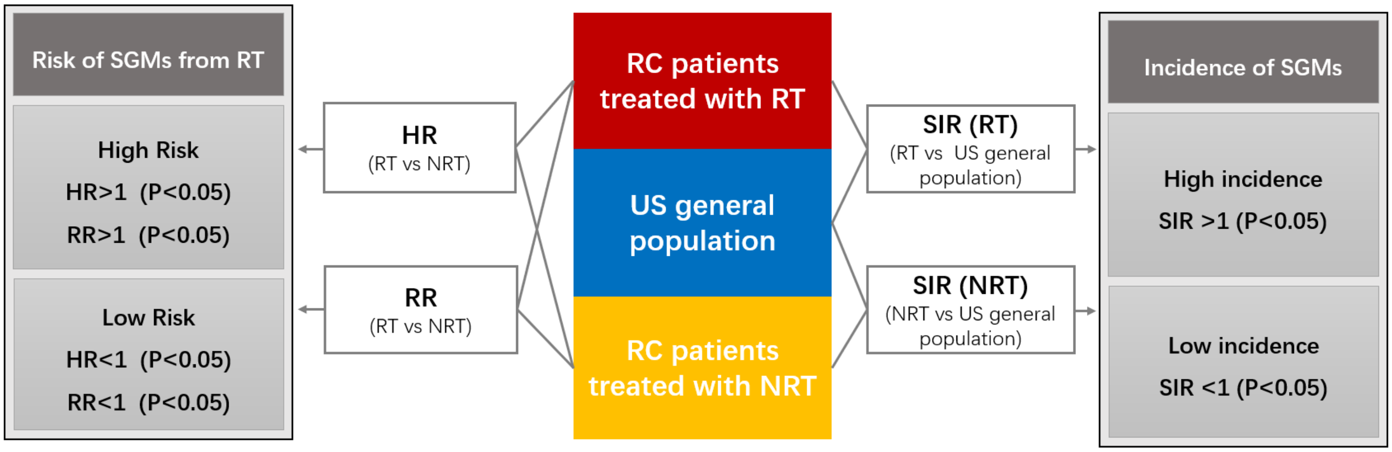

eFigure 2. Statistical Methods Annotation. Fine-Gray competing risk regression was used to calculate HR and 95% CIs of SGM in RC patients who underwent RT compared with those who did not undergo RT. The RR was estimated by using Poisson regression analysis with relative risks and 95% CIs of SGM in RC patients who underwent RT compared with those who did not undergo RT. The RR was adjusted for age at RC diagnosis and calendar year of RC diagnosis. In our study, a **high risk** of developing SGMs from RT required both adjusted HR>1 and adjusted RR>1, and a **low risk** of developing SGMs from RT required both adjusted HR<1 and adjusted RR<1. Poisson regression analysis was used to calculate the SIR and 95% CIs. SIR was defined as the ratio of observed SGMs among RC survivors to the incidence of GMs in the US general population. The SIR was adjusted for age at RC diagnosis and calendar year of RC diagnosis. SIR> 1 indicated a **high incidence** of SGMs, and SIR<1 indicated a **low incidence** of SGMs.

Abbreviations: RC, rectal cancer; RT, radiation therapy; NRT, no radiation therapy; CI, confidence interval; SGM, second gynecological malignancy; SIR, standardized incidence ratio; HR, hazard ratio; RR, radiation-attributed risk.

© 2021 Guan X et al. *JAMA Network Open.*

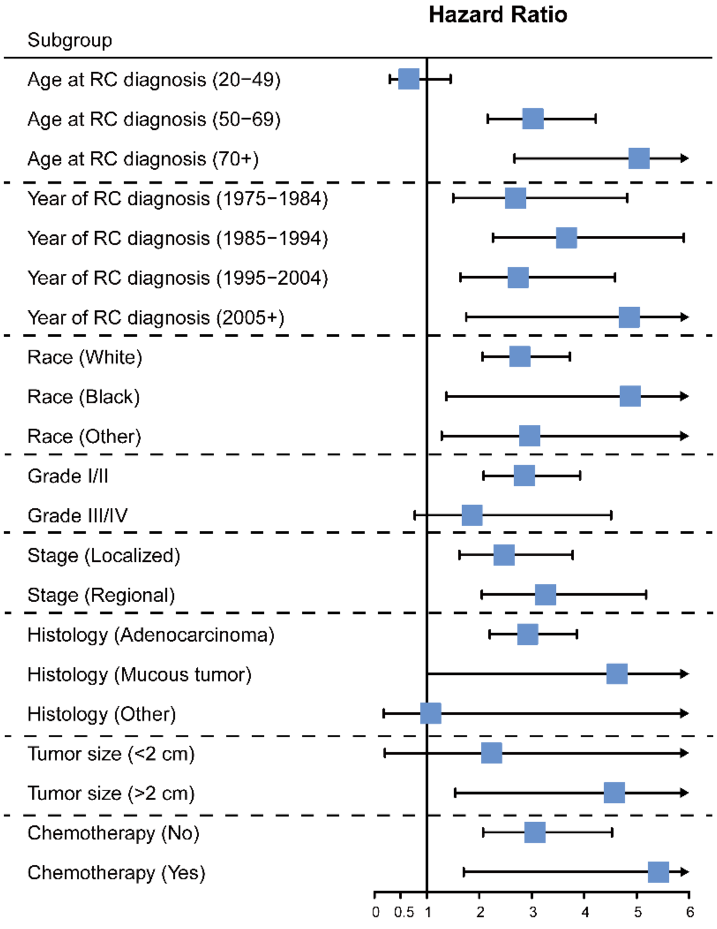

eFigure 3. Subgroup analyses of competing risk regression for the risk of developing corpus uteri cancer.

© 2021 Guan X et al. *JAMA Network Open.*

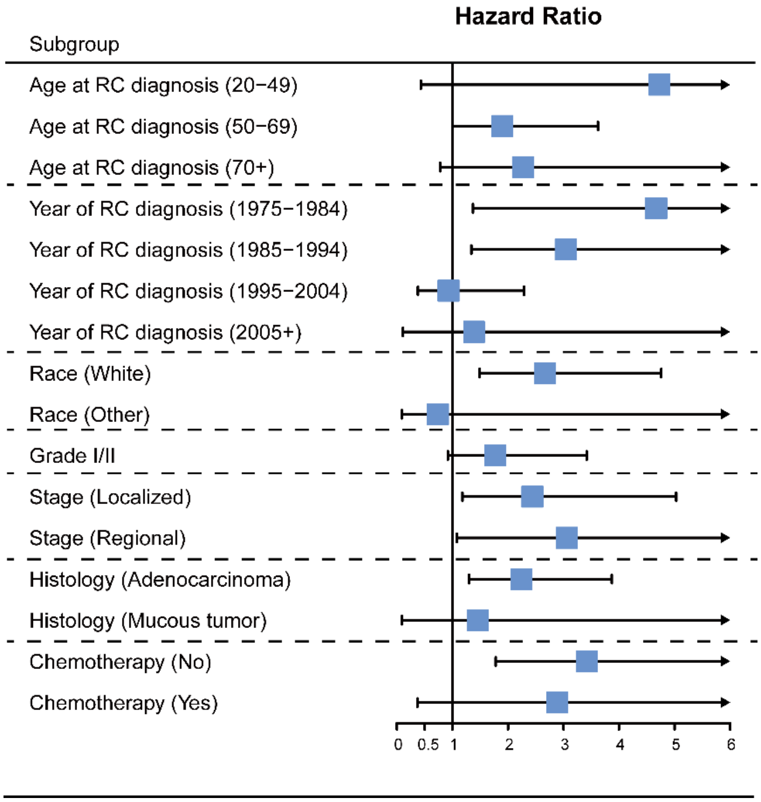

eFigure 4. Subgroup analyses of competing risk regression for the risk of developing ovarian cancer.

© 2021 Guan X et al. *JAMA Network Open.*

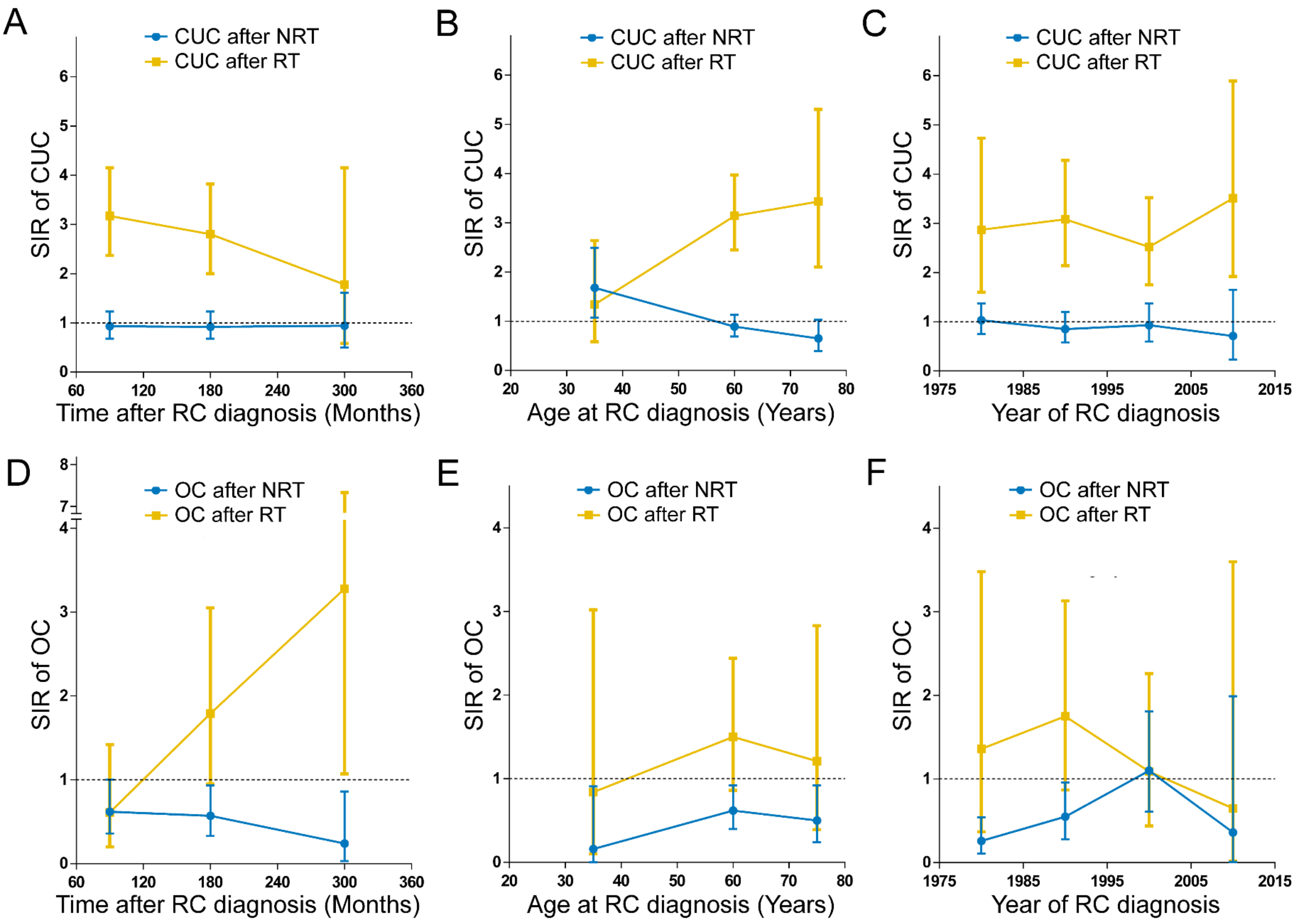

eFigure 5. (A) Dynamic standardized incidence ratio (SIR) for CUC in latency-SIR plot; (B) Dynamic SIR for CUC in age-RR plot; (C) Dynamic SIR for CUC in diagnosis time-RR plot; (D) Dynamic SIR for OC in latency-RR plot; (E) Dynamic SIR for OC in age-RR plot; (F) Dynamic SIR for OC in diagnosis time-RR plot.

NOTE. (A.B.C.D.E.F) Adjusted SIRs and 95% CIs of developing CUC and OC in patients treated with RT versus the US general population are plotted, as well as patients treated without RT versus the US general population, and the incidence in the background US population is represented by the gray line (at y=1). The detailed data of SIRs are shown in the supplementary data.

Abbreviations: HR, hazard ratio; RC, rectal cancer; CI, confidence interval.

© 2021 Guan X et al. *JAMA Network Open.*

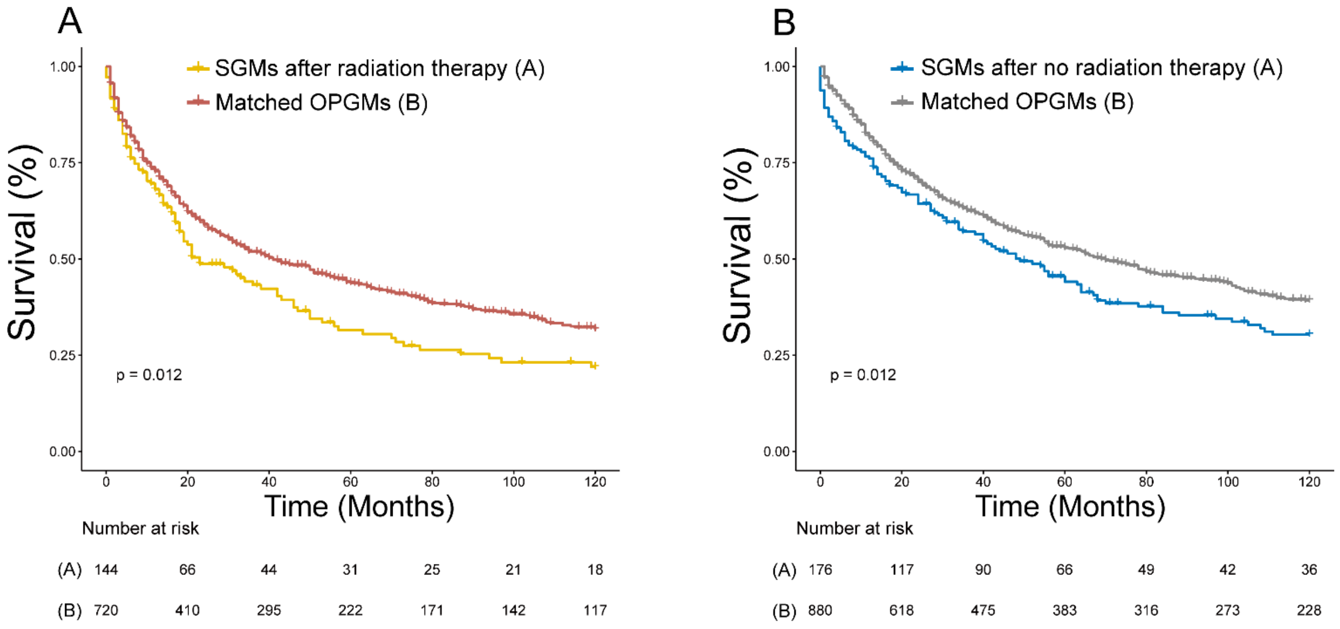

eFigure 6. (A) Survival comparison between RC patients who developed SGMs after RT and matched OPGMs; (B) Survival comparison between RC patients who developed SGMs after NRT and matched OPGMs. RC patients who developed SGMs versus matched OPGMs, with a 1:5 PSM ratio of SGMs to OPGMs. The matched variables for PSM included age at GM diagnosis, year of GM diagnosis, race, stage of GM and type of treatment for GM.

Abbreviations: RC, rectal cancer; RT, radiation therapy; NRT, no radiation therapy; SGM, second gynecological malignancy; GM, gynecological malignancy; OPGM, only primary gynecological malignancy.

© 2021 Guan X et al. *JAMA Network Open.*

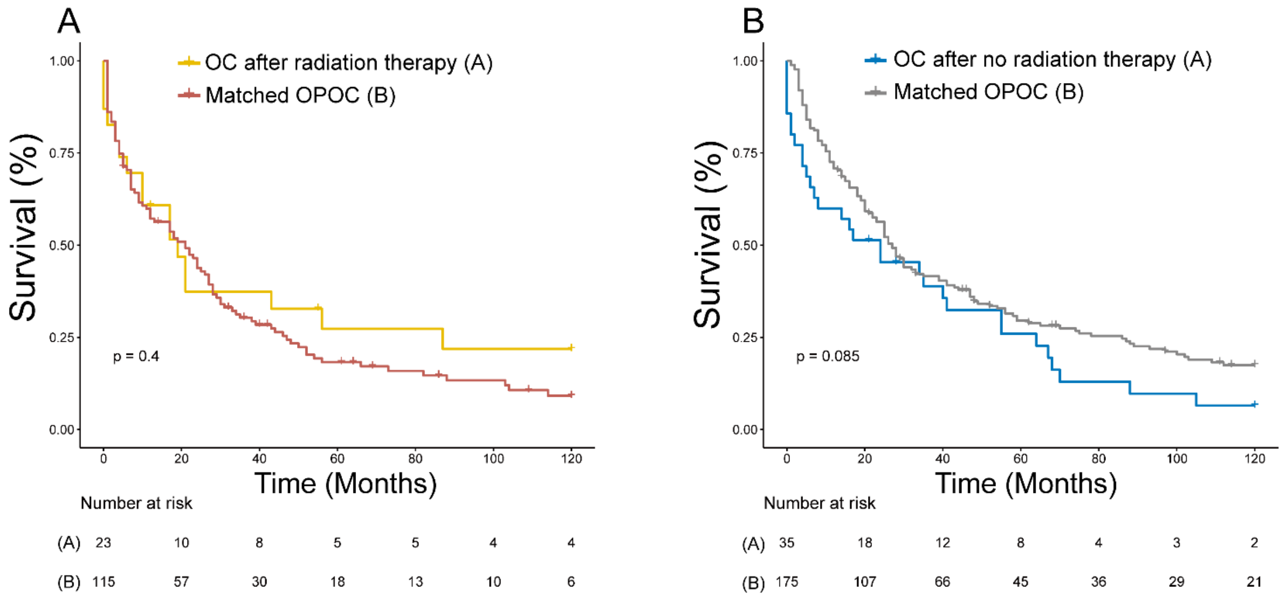

eFigure 7. (A) Survival comparison between RC patients who developed OC after RT and matched OPOC patients; (B) Survival comparison between RC patients who developed OC after NRT and matched OPOC patients. RC patients who developed OC versus matched OPOC patients, with a PSM ratio of 1:5 for OC versus OPOC patients. The matched variables for PSM included age at GM diagnosis, year of GM diagnosis, race, stage of GM and type of treatment for GM.

Abbreviations: RC, rectal cancer; RT, radiation therapy; NRT, no radiation therapy; GM, gynecological malignancy; OC, ovarian cancer; OPOC, only primary ovarian cancer.

© 2021 Guan X et al. *JAMA Network Open.*

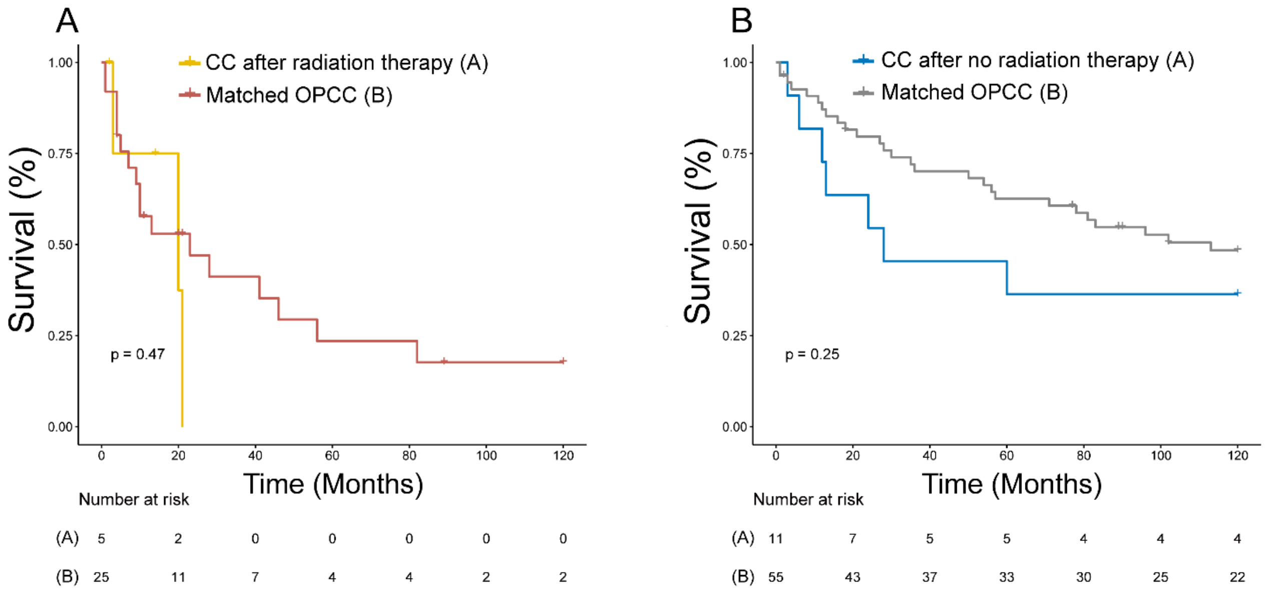

eFigure 8. (A) Survival comparison between RC patients who developed CC after RT and matched OPCC patients; (B) Survival comparison between RC patients who developed CC after NRT and matched OPCC patients. RC patients who developed CC versus matched OPCC patients, with a PSM ratio of 1:5 for CC versus OPCC patients. The matched variables for PSM included age at GM diagnosis, year of GM diagnosis, race, stage of GM and type of treatment for GM.

Abbreviations: RC, rectal cancer; RT, radiation therapy; NRT, no radiation therapy; GM, gynecological malignancy; CC, cervical cancer; OPCC, only primary cervical cancer.

© 2021 Guan X et al. *JAMA Network Open.*

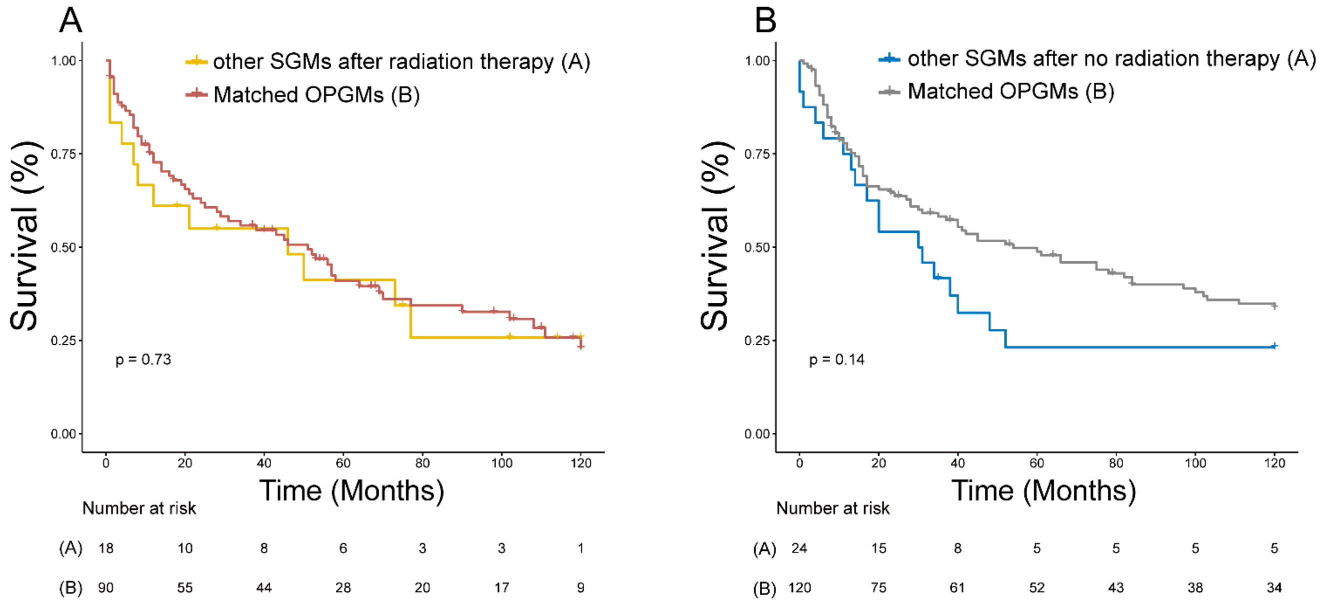

eFigure 9. Survival comparison between RC patients who developed other types of SGMs after RT and matched OPGM patients; (B) Survival comparison between RC patients who developed other types of SGMs after NRT and matched OPGM patients. RC patients who developed SGMs versus matched OPGM patients, with a PSM of 1:5 for SGM versus OPGM patients. The matched variables for PSM included age at GM diagnosis, year of GM diagnosis, race, stage of GM and type of treatment for GM.

Abbreviations: RC, rectal cancer; RT, radiation therapy; NRT, no radiation therapy; SGM, second gynecological malignancy; GM, gynecological malignancy; OPGM, only primary gynecological malignancy.

eTable 1. The Description of Tumor Stage of Rectal Cancer in SEER Program.

Tumor Stage	Description
In situ	A noninvasive neoplasm; a tumor which has not penetrated the basement membrane nor extended beyond the epithelial tissue. Some synonyms are intraepithelial (confined to epithelial tissue), noninvasive and noninfiltrating.
Localized Stage	An invasive neoplasm confined entirely to the organ of origin. It may include intraluminal extension where specified. For example, for colon, intraluminal extension limited to immediately contiguous segments of the large bowel is localized, if no lymph nodes are involved. Localized may exclude invasion of the serosa because of the poor survival of the patient once the serosa is invaded.
Regional Stage	A neoplasm that has extended (1) beyond the limits of the organ of origin directly into surrounding organs or tissues; (2) into regional lymph nodes by way of the lymphatic system; (3) by a combination of extension and regional lymph nodes.
Distant Stage	A neoplasm that has spread to parts of the body remote from the primary tumor either by direct extension or by discontinuous metastasis (implantation or seeding) to distant organs, issues, or via the lymphatic system to distant lymph nodes.

NOTE. The description of tumor stage of rectal cancer in SEER Program was derived from SEER official website (https://seer.cancer.gov/).

eTable 2. Comparisons of Baseline Characteristics of RC Patients Who Developed SGMs by Treatment Type

Characteristic	NRT (n=176)	RT (n=144)	P
Median age at RC diagnosis, (IQR), years	61 (53-69)	60 (54-68)	0.934[a]
Age at RC diagnosis, No. (%), years			0.216[b]
20-49	27 (15.3)	14 (9.8)	
50-69	110 (62.5)	102 (70.8)	
≥ 70	39 (22.2)	28 (19.4)	
Median year of RC diagnosis (IQR)	1988 (1982-1996)	1994 (1987-2002)	< 0.001[a]
Year of RC diagnosis, No. (%)			< 0.001[b]
1975-1984	67 (38.1)	21 (14.6)	
1985-1994	56 (31.8)	53 (36.8)	
1995-2004	46 (26.1)	49 (34)	
≥ 2005	7 (4)	21 (14.6)	
Race, No. (%)			0.621[b]
White	146 (83)	125 (86.8)	
Black	12 (6.8)	7 (13.5)	
Other	18 (10.2)	12 (8.3)	
Tumor grade, No. (%)			0.128[b]
Grade I/II	117 (66.5)	108 (75)	
Grade III/IV	16 (9.1)	14 (9.7)	
Unknown	43 (24.4)	22 (15.3)	
Tumor stage, No. (%)			0.057[b]
Localized	112 (63.6)	106 (73.6)	
Regional	64 (36.4)	38 (26.4)	
Tumor histology, %			0.260[b]
Adenocarcinoma	168 (95.5)	131 (91)	

© 2021 Guan X et al. *JAMA Network Open.*

Mucous tumor	5 (2.8)	9 (6.2)	
Other	3 (1.7)	4 (2.8)	
Tumor size, No. (%), cm			< 0.001[b]
< 2	2 (1.1)	2 (1.4)	
≥ 2	5 (2.8)	23 (16)	
Unknown	169 (96)	119 (82.6)	
Chemotherapy, No. (%)			< 0.001[b]
No	169 (96)	53 (36.8)	
Yes	7 (4)	91 (63.2)	
Median follow-up time of RC, (IQR), months	189 (135-266)	163 (114-235)	0.011[a]
Total person-years at risk	1,674	2,026	
Median latency between RC and SGM, (IQR), months	124 (93-168)	121 (93-175)	0.831[a]

NOTE. P values were calculated using the Mann-Whitney U test (a) for continuous variables and χ^2 test (b) for categorical variables.

Abbreviations: RC, rectal cancer; RT, radiation therapy; NRT, no radiation therapy; IQR, interquartile ratio; SGM, second gynecological malignancy.

© 2021 Guan X et al. *JAMA Network Open.*

eTable 3. Univariable and Multivariable Competing Risk Regression Analysis of Risk of Developing Combined SGMs in RC Patients

Characteristic	Univariable Competing Risk Regression		Multivariable Competing Risk Regression	
	HR (95% Cl)	P	HR (95% Cl)	P
Age at RC diagnosis, per year	0.98 (0.97-0.98)	<0.001	0.98 (0.97-0.99)	<0.001
Year of RC diagnosis, per year	0.99 (0.98-1.01)	0.670	-	-
Race				
White	1		-	
Black	0.87 (0.55-1.39)	0.570	-	-
Other	1.17 (0.80-1.71)	0.410	-	-
Tumor grade				
Grade I/II	1		-	
Grade III/IV	0.96 (0.66-1.41)	0.840	-	-
Unknown	0.94 (0.71-1.23)	0.640	-	-
Tumor stage				
Localized	1		1	
Regional	1.43 (1.15-1.78)	0.002	1.02 (0.79-1.31)	0.890
Tumor histology				
Adenocarcinoma	1		-	
Mucous tumor	0.99 (0.58-1.69)	0.980	-	-
Other	1.57 (0.75-3.33)	0.240	-	-
Tumor size (cm)				
<2	1		-	
≥2	2.22 (0.78-6.33)	0.140	-	-
Unknown	1.71 (0.64-4.61)	0.290	-	-
Chemotherapy				
No	1		1	
Yes	1.67 (1.32-2.12)	<0.001	0.68 (0.50-0.93)	0.016
Radiation therapy				
No	1		1	
Yes	2.59 (2.07-3.23)	<0.001	2.99 (2.23-4.02)	<0.001

NOTE. Fine-Gray competing risk regression analyses are used to calculate the hazard ratios (HRs) and 95% confidence intervals (CIs) for SGM in rectal cancer (RC) patients treated with RT versus patients not treated with RT. Covariables that are significant in univariable competing risk regression analysis ($P < 0.05$) are included in the multivariable analysis.

Abbreviations: HR, hazard ratio; CI, confidence interval; RC, rectal cancer.

© 2021 Guan X et al. *JAMA Network Open.*

eTable 4. Univariable and Multivariable Competing Risk Regression Analysis of Risk of Developing Corpus Uteri Cancer in RC Patients

Characteristic	Univariable Competing Risk Regression		Multivariable Competing Risk Regression	
	HR (95% Cl)	P	HR (95% Cl)	P
Age at RC diagnosis, per year	0.97 (0.96-0.98)	<0.001	0.98 (0.96-0.99)	<0.001
Year of RC diagnosis, per year	0.99 (0.98-1.01)	0.920	-	-
Race				
White	1		-	
Black	0.64 (0.33-1.26)	0.200	-	-
Other	1.33 (0.86-2.08)	0.200	-	-
Tumor grade				
Grade I/II	1		-	
Grade III/IV	0.89 (0.56-1.44)	0.640	-	-
Unknown	0.72 (0.50-1.04)	0.082	-	-
Tumor stage				
Localized	1		1	
Regional	1.6 (1.22-2.11)	0.001	1.10 (0.81-1.50)	0.530
Tumor histology				
Adenocarcinoma	1		-	
Mucous tumor	0.88 (0.43-1.78)	0.710	-	-
Other	1.73 (0.72-4.19)	0.220	-	-
Tumor size (cm)				
<2	1		-	
≥2	2.19 (0.65-7.34)	0.210	-	-
Unknown	1.39 (0.44-4.38)	0.570	-	-
Chemotherapy				
No	1		1	
Yes	1.87 (1.40-2.50)	<0.001	0.71 (0.49-1.02)	0.064
Radiation therapy				
No	1		1	
Yes	2.87 (2.19-3.77)	<0.001	3.06 (2.14-4.37)	<0.001

NOTE. Fine-Gray competing risk regression analyses are used to calculate the hazard ratios (HRs) and 95% confidence intervals (CIs) for SGM in rectal cancer (RC) patients treated with RT versus patients not treated with RT. Covariables that are significant in univariable competing risk regression analysis (P< 0.05) are included in the multivariable analysis.

Abbreviations: HR, hazard ratio; CI, confidence interval; RC, rectal cancer.

© 2021 Guan X et al. *JAMA Network Open.*

eTable 5. Univariable and Multivariable Competing Risk Regression Analysis of Risk of Developing Ovarian Cancer in RC Patients

Characteristic	Univariable Competing Risk Regression		Multivariable Competing Risk Regression	
	HR (95% Cl)	P	HR (95% Cl)	P
Age at RC diagnosis, per year	0.99 (0.98-1.01)	0.270	-	-
Year of RC diagnosis, per year	0.99 (0.97-1.02)	0.820	-	-
Race				
White	1		-	
Black	1.32 (0.52-3.32)	0.560	-	-
Other	1.11 (0.44-2.80)	0.820	-	-
Tumor grade				
Grade I/II	1		-	
Grade III/IV	0.56 (0.17-1.80)	0.330	-	-
Unknown	1.33 (0.75-2.39)	0.330	-	-
Tumor stage				
Localized	1		-	
Regional	0.86 (0.49-1.50)	0.590	-	-
Tumor histology				
Adenocarcinoma	1		-	
Mucous tumor	0.77 (0.19-3.16)	0.720	-	-
Other	1.22 (0.17-8.82)	0.850	-	-
Tumor size (cm)				
<2	-	-	-	
≥2	1		-	-
Unknown	1.71 (0.64-4.61)	0.290	-	-
Chemotherapy				
No	1		-	
Yes	1.00 (0.53-1.90)	0.990	-	-
Radiation therapy				
No	1		1	
Yes	2.08 (1.22-3.56)	0.007	2.08 (1.22-3.56)	0.007

NOTE. Fine-Gray competing risk regression analyses are used to calculate the hazard ratios (HRs) and 95% confidence intervals (CIs) for SGM in rectal cancer (RC) patients treated with RT versus patients not treated with RT. Covariables that are significant in univariable competing risk regression analysis (P< 0.05) are included in the multivariable analysis.

Abbreviations: HR, hazard ratio; CI, confidence interval; RC, rectal cancer.

© 2021 Guan X et al. *JAMA Network Open.*

eTable 6. Univariable and Multivariable Competing Risk Regression Analysis of Risk of Developing Cervical Cancer in RC Patients

Characteristic	Univariable Competing Risk Regression		Multivariable Competing Risk Regression	
	HR (95% Cl)	P	HR (95% Cl)	P
Age at RC diagnosis, per year	0.97 (0.94-1.01)	0.100	-	-
Year of RC diagnosis, per year	0.99 (0.94-1.04)	0.640	-	-
Race				
White	1		-	
Black	1.97 (0.43-8.74)	0.390	-	-
Other	1.64 (0.37-7.26)	0.520	-	-
Tumor grade				
Grade I/II	1		-	
Grade III/IV	0.72 (0.09-5.60)	0.750	-	-
Unknown	1.61 (0.55-4.70)	0.390	-	-
Tumor stage				
Localized	1		-	
Regional	1.13 (0.41-3.21)	0.810	-	-
Tumor histology				
Adenocarcinoma	1		-	
Mucous tumor	0.90 (0.21-3.81)	0.890	-	-
Other	-	-	-	-
Tumor size (cm)				
<2	-		-	
≥2	1		-	
Unknown	0.92 (0.40-2.14)	0.850	-	-
Chemotherapy				
No	1		-	
Yes	0.77 (0.22-2.68)	0.680	-	-
Radiation therapy				
No	1		-	
Yes	1.33 (0.46-3.83)	0.600	-	-

NOTE. Fine-Gray competing risk regression analyses are used to calculate the hazard ratios (HRs) and 95% confidence intervals (CIs) for SGM in rectal cancer (RC) patients treated with RT versus patients not treated with RT. Covariables that are significant in univariable competing risk regression analysis ($P < 0.05$) are included in the multivariable analysis.

Abbreviations: HR, hazard ratio; CI, confidence interval; RC, rectal cancer.

© 2021 Guan X et al. *JAMA Network Open.*

eTable 7. Univariable and Multivariable Competing Risk Regression Analysis of Risk of Developing Other SGMs in RC Patients

Characteristic	Univariable Competing Risk Regression		Multivariable Competing Risk Regression	
	HR (95% Cl)	P	HR (95% Cl)	P
Age at RC diagnosis, per year	0.99 (0.97-1.01)	0.240	-	-
Year of RC diagnosis, per year	0.99 (0.96-1.02)	0.450	-	-
Race				
White	1		-	
Black	0.99 (0.31-3.19)	0.980	-	-
Other	0.28 (0.03-2.02)	0.210	-	-
Tumor grade				
Grade I/II	1		1	
Grade III/IV	2.41 (1.08-5.36)	0.031	2.14 (0.93-4.92)	0.074
Unknown	1.35 (0.65-2.83)	0.420	-	-
Tumor stage				
Localized	1		-	
Regional	1.57 (0.86-2.89)	0.140	-	-
Tumor histology				
Adenocarcinoma	1		-	
Mucous tumor	1.72 (0.53-5.58)	0.360	-	-
Other	3.62 (0.88-15.00)	0.076	-	-
Tumor size (cm)				
<2	1		-	
≥2	-	-	-	-
Unknown	1.14 (0.55-2.35)	0.730	-	-
Chemotherapy				
No	1		1	
Yes	2.1 (1.11-3.96)	0.022	1.28 (0.45-3.65)	0.640
Radiation therapy				
No	1		1	
Yes	2.35 (1.27-4.37)	0.006	2.02 (0.71-5.57)	0.180

NOTE. Fine-Gray competing risk regression analyses are used to calculate the hazard ratios (HRs) and 95% confidence intervals (CIs) for SGM in rectal cancer (RC) patients treated with RT versus patients not treated with RT. Covariables that are significant in univariable competing risk regression analysis (P< 0.05) are included in the multivariable analysis.

Abbreviations: HR, hazard ratio; CI, confidence interval; RC, rectal cancer.

© 2021 Guan X et al. *JAMA Network Open.*

eTable 8. Subgroup Analyses of Hazard Ratios of Developing Combined SGMs in RC Patients who Received RT versus RC Patients who did not Received RT.

Subgroup	RT (No. of events/total No.)	NRT (No. of events/total No.)	HR (95% CI)	P
Age at RC diagnosis (20-49)	14/1047	27/1767	1.02 (0.54-1.95)	0.950
Age at RC diagnosis (50-69)	102/2886	110/7230	2.64 (2.02-3.46)	<0.001
Age at RC diagnosis (70+)	28/1377	39/5835	3.28 (2.01-5.31)	<0.001
Year of RC diagnosis (1975-1984)	21/503	67/4095	2.59 (1.58-4.22)	<0.001
Year of RC diagnosis (1985-1994)	53/1228	56/4063	3.17 (2.18-4.61)	<0.001
Year of RC diagnosis (1995-2004)	49/1984	46/3983	2.15 (1.44-3.21)	<0.001
Year of RC diagnosis (2005+)	21/1595	7/2691	5.21 (2.22-12.20)	<0.001
Race (White)	125/4449	146/12353	2.70 (2.12-3.43)	<0.001
Race (Black)	7/352	12/1170	2.20 (0.88-5.51)	0.093
Race (Other)	12/509	8/1309	1.95 (0.94-4.04)	0.074
Grade I/II	108/3992	117/9899	2.61 (2.00-3.39)	<0.001
Grade III/IV	14/777	16/1141	1.43 (0.70-2.95)	0.330
Stage (Localized)	46/1742	137/11393	2.47 (1.77-3.45)	<0.001
Stage (Regional)	98/3568	39/3439	2.84 (1.96-4.12)	<0.001
Histology (Adenocarcinoma)	131/4831	168/14181	2.60 (2.07-3.27)	<0.001
Histology (Mucous tumor)	9/360	5/490	2.72 (0.94-7.92)	0.066
Histology (Other)	4/119	3/161	2.04 (0.45-9.21)	0.350
Tumor size (<2 cm)	2/191	2/827	4.45 (0.63-31.40)	0.130
Tumor size (>2 cm)	23/1306	5/1323	4.95 (1.88-13.00)	0.001
Chemotherapy (No)	53/1293	169/13892	3.08 (2.26-4.19)	<0.001

© 2021 Guan X et al. *JAMA Network Open.*

Chemotherapy (Yes)	91/4017	7/940	3.29 (1.52-7.11)	0.003

NOTE. Fine-Gray competing risk regression analyses are used to calculate the hazard ratios (HRs) and 95% confidence intervals (CIs) for SGM in rectal cancer (RC) patients treated with radiation therapy (RT) versus patients not treated with RT.

Abbreviations: HR, hazard ratio; CI, confidence interval; RT, radiation therapy; NRT, no radiation therapy; RC, rectal cancer.

© 2021 Guan X et al. *JAMA Network Open.*

eTable 9. Subgroup Analyses of Hazard Ratios of Developing Corpus Uteri Cancer in RC Patients who Received RT versus Those who did not Receive RT.

Subgroup	RT (No. of events/total No.)	NRT (No. of events/total No.)	HR (95% CI)	P
Age at RC diagnosis (20-49)	8/1047	23/1767	0.66 (0.29-1.45)	0.300
Age at RC diagnosis (50-69)	70/2886	65/7230	3.02 (2.16-4.22)	0.001
Age at RC diagnosis (70+)	20/1377	18/5835	5.05 (2.67-9.53)	0.001
Year of RC diagnosis (1975-1984)	15/503	44/4095	2.69 (1.50-4.82)	0.001
Year of RC diagnosis (1985-1994)	35/1228	32/4063	3.66 (2.26-5.90)	0.001
Year of RC diagnosis (1995-2004)	34/1984	25/3983	2.74 (1.64-4.59)	0.001
Year of RC diagnosis (2005+)	14/1595	5/2691	4.86 (1.75-13.50)	0.002
Race (White)	82/4449	91/12353	2.77 (2.06-3.73)	0.001
Race (Black)	5/352	4/1170	4.88 (1.37-17.30)	0.014
Race (Other)	11/509	11/1309	2.96 (1.28-6.81)	0.011
Grade I/II	77/3992	74/9899	2.86 (2.08-3.92)	0.001
Grade III/IV	10/777	9/1141	1.86 (0.77-4.51)	0.170
Stage (Localized)	28/1742	82/11393	2.47 (1.62-3.78)	0.001
Stage (Regional)	70/3568	24/3439	3.26 (2.05-5.18)	0.001
Histology (Adenocarcinoma)	90/4831	102/14181	2.92 (2.20-3.86)	0.001
Histology (Mucous tumor)	6/360	2/490	4.63 (1.00-21.50)	0.050
Histology (Other)	2/119	2/161	1.07 (0.18-6.21)	0.940
Tumor size (<2 cm)	1/191	2/827	2.23 (0.20-24.60)	0.510
Tumor size (>2 cm)	17/1306	4/1323	4.58 (1.54-13.60)	0.006
Chemotherapy (No)	33/1293	103/13892	3.06 (2.07-4.53)	0.001

© 2021 Guan X et al. *JAMA Network Open.*

Chemotherapy (Yes)	65/4017	3/940	5.42 (1.70-17.30)	0.004

NOTE. Fine-Gray competing risk regression analyses are used to calculate the hazard ratios (HRs) and 95% confidence intervals (CIs) for SGM in rectal cancer (RC) patients treated with radiation therapy (RT) versus patients not treated with RT.

Abbreviations: HR, hazard ratio; CI, confidence interval; RT, radiation therapy; NRT, no radiation therapy; RC, rectal cancer.

© 2021 Guan X et al. *JAMA Network Open.*

eTable 10. Subgroup Analyses of Hazard Ratios of Developing Ovarian Cancer in RC Patients who Received RT versus Those who did not Receive RT.

Subgroup	RT (No. of events/total No.)	NRT (No. of events/total No.)	HR (95% CI)	P
Age at RC diagnosis (20-49)	2/1047	1/1767	4.73 (0.43-51.80)	0.200
Age at RC diagnosis (50-69)	16/2886	24/7230	1.90 (1.00-3.62)	0.051
Age at RC diagnosis (70+)	5/1377	10/5835	2.27 (0.78-6.60)	0.130
Year of RC diagnosis (1975-1984)	4/503	7/4095	4.67 (1.37-15.90)	0.014
Year of RC diagnosis (1985-1994)	11/1228	12/4063	3.04 (1.34-6.89)	0.008
Year of RC diagnosis (1995-2004)	7/1984	15/3983	0.93 (0.38-2.29)	0.880
Year of RC diagnosis (2005+)	1/1595	1/2691	1.39 (0.11-26.90)	0.710
Race (White)	22/4449	26/12353	2.67 (1.49-4.76)	0.001
Race (Other)	1/509	4/1309	0.74 (0.09-6.42)	0.780
Grade I/II	15/3992	24/9899	1.77 (0.92-3.42)	0.089
Stage (Localized)	10/1742	30/11393	2.44 (1.18-5.03)	0.016
Stage (Regional)	13/3568	5/3439	3.06 (1.08-8.73)	0.036
Histology (Adenocarcinoma)	22/4831	33/14181	2.24 (1.30-3.87)	0.003
Histology (Mucous tumor)	1/360	1/490	1.46 (0.09-23.50)	0.760
Chemotherapy (No)	12/1293	34/13892	3.42 (1.78-6.55)	0.001
Chemotherapy (Yes)	11/4017	1/940	2.89 (0.37-22.60)	0.310

NOTE. Fine-Gray competing risk regression analyses are used to calculate the hazard ratios (HRs) and 95% confidence intervals (CIs) for SGM in rectal cancer (RC) patients treated with radiation therapy (RT) versus patients not treated with RT.

Abbreviations: HR, hazard ratio; CI, confidence interval; RT, radiation therapy; NRT, no radiation therapy; RC, rectal cancer.

© 2021 Guan X et al. *JAMA Network Open.*

eTable 11. Subgroup Analyses of Hazard Ratios of Developing Cervical Cancer in RC Patients who Received RT versus Those who did not Receive RT.

Subgroup	RT (No. of events/total No.)	NRT (No. of events/total No.)	HR (95% CI)	P
Age at RC diagnosis (20-49)	1/1047	2/1767	0.87 (0.08-9.43)	0.910
Age at RC diagnosis (50-69)	4/2886	5/7230	2.09 (0.56-7.79)	0.270
Year of RC diagnosis (1985-1994)	1/1228	6/4063	0.55 (0.07-4.57)	0.580
Year of RC diagnosis (1995-2004)	1/1984	1/3983	2.01 (0.13-32.10)	0.620
Race (White)	5/4449	7/12353	2.09 (0.66-6.56)	0.210
Grade I/II	4/3992	6/9899	1.74 (0.49-6.17)	0.390
Stage (Localized)	1/1742	9/11393	0.76 (0.10-6.03)	0.800
Stage (Regional)	4/3568	2/3439	2.03 (0.38-11.00)	0.410
Histology (Adenocarcinoma)	4/4831	11/14181	1.13 (0.36-3.54)	0.840
Chemotherapy (No)	2/1293	11/13892	1.86 (0.41-8.40)	0.420

NOTE. Fine-Gray competing risk regression analyses are used to calculate the hazard ratios (HRs) and 95% confidence intervals (CIs) for SGM in rectal cancer (RC) patients treated with radiation therapy (RT) versus patients not treated with RT.

Abbreviations: HR, hazard ratio; CI, confidence interval; RT, radiation therapy; NRT, no radiation therapy; RC, rectal cancer.

© 2021 Guan X et al. *JAMA Network Open.*

eTable 12. Subgroup Analyses of Hazard Ratios of Developing Other SGMs in RC Patients who Received RT versus Those who did not Receive RT.

Subgroup	RT (No. of events/total No.)	NRT (No. of events/total No.)	HR (95%CI)	P
Age at RC diagnosis (20-49)	3/1047	1/1767	6.16 (0.72-52.5)	0.096
Age at RC diagnosis (50-69)	12/2886	16/7230	2.11 (1.00-4.53)	0.050
Age at RC diagnosis (70+)	3/1377	7/5835	1.95 (0.50-7.55)	0.340
Year of RC diagnosis (1975-1984)	2/503	12/4095	1.36 (0.30-6.05)	0.690
Year of RC diagnosis (1985-1994)	6/1228	6/4063	3.31 (1.07-10.30)	0.038
Year of RC diagnosis (1995-2004)	7/1984	5/3983	2.81 (0.89-8.88)	0.078
Year of RC diagnosis (2005+)	3/1595	1/2691	5.38 (0.54-49.60)	0.150
Race (White)	16/4449	22/12353	2.29 (1.19-4.40)	0.013
Race (Black)	2/352	1/1170	1.94 (0.63-76.20)	0.110
Grade I/II	12/3992	12/9899	2.84 (1.26-6.41)	0.012
Grade III/IV	1/777	7/1141	0.23 (0.03-1.94)	0.180
Stage (Localized)	7/1742	16/11393	3.13 (1.27-7.71)	0.013
Stage (Regional)	11/3568	8/3439	1.58 (0.63-3.94)	0.330
Histology (Adenocarcinoma)	15/4831	12/14181	2.26 (1.16-4.39)	0.017
Histology (Mucous tumor)	1/360	2/490	0.73 (0.07-7.89)	0.800
Tumor size (>2 cm)	3/1306	1/1323	3.16 (0.33-30.50)	0.320
Chemotherapy (No)	6/1293	21/13892	2.77 (1.13-6.80)	0.026
Chemotherapy (Yes)	12/4017	3/940	1.01 (0.28-3.65)	0.980

NOTE. Fine-Gray competing risk regression analyses are used to calculate the hazard ratios (HRs) and 95% confidence intervals (CIs) for SGM in rectal cancer (RC) patients treated with radiation therapy (RT) versus patients not treated with RT.

© 2021 Guan X et al. *JAMA Network Open.*

Abbreviations: HR, hazard ratio; CI, confidence interval; RT, radiation therapy; NRT, no radiation therapy; RC, rectal cancer.

© 2021 Guan X et al. *JAMA Network Open.*

eTable 13. Radiation-attributed Risk of Combined SGMs by Age at RC Diagnosis, Latency and Year of RC Diagnosis.

Characteristic	RR (95% Cl)	P	Adjusted RR (95% Cl)	P
Age at RC diagnosis, years	2.52 (2.02-3.14)	<0.001	2.85 (2.27-3.58)	<0.001
20-49	1.01 (0.53-1.92)	0.04	1.18 (0.61-2.29)	0.620
50-69	2.78 (2.12-3.64)	<0.001	3.17 (2.40-4.20)	<0.001
≥ 70	3.26 (2.01-5.30)	<0.001	3.20 (1.93-5.30)	<0.001
Latency between RC and SGM, months	2.21 (1.77-2.75)	<0.001	2.54 (2.02-3.21)	<0.001
60-119	1.93 (1.40-2.65)	<0.001	2.26 (1.62-3.16)	<0.001
120-239	2.33 (1.66-3.27)	<0.001	2.67 (1.88-3.81)	<0.001
240-360	3.51 (1.70-7.23)	0.001	3.83 (1.81-8.12)	<0.001
Year of RC diagnosis	2.81 (2.23-3.53)	<0.001	2.55 (2.04-3.19)	<0.001
1975-1984	2.67 (1.63-4.35)	<0.001	2.50 (1.53-4.11)	<0.001
1985-1994	3.19 (2.19-4.64)	<0.001	3.13 (2.14-4.57)	<0.001
1995-2004	2.16 (1.44-3.23)	<0.001	2.20 (1.46-3.30)	<0.001
≥ 2005	5.16 (2.19-12.14)	<0.001	5.34 (2.27-12.59)	<0.001

NOTE. Poisson regression analyses were used to calculate the radiation-attributed risk (RR) and 95% confidence intervals (CIs) of SGMs for patients with radiation therapy versus patients without radiation therapy. Adjusted RRs were stratified by age at RC diagnosis and calendar year of RC diagnosis. A determination of statistical significance of RRs was based on P<0.05 (two sided).

Abbreviations: RC, rectal cancer; RR, radiation-attributed risk; SGM, second gynecological malignancy.

© 2021 Guan X et al. *JAMA Network Open.*

eTable 14. Radiation-attributed Risk of Corpus Uteri Cancer by Age at RC Diagnosis, Latency and Year of RC Diagnosis.

Characteristic	RR (95% Cl)	P	Adjusted RR (95% Cl)	P
Age at RC diagnosis, years	2.78 (2.11-3.66)	<0.001	3.27 (2.46-4.36)	<0.001
20-49	0.68 (0.30-1.50)	0.337	0.79 (0.35-1.79)	0.571
50-69	3.21 (2.29-4.49)	<0.001	3.74 (2.63-5.32)	<0.001
≥ 70	5.05 (2.67-9.54)	<0.001	5.13 (2.64-9.97)	<0.001
Latency between RC and SGM, months	2.49 (1.89-3.27)	<0.001	2.84 (2.13-3.80)	<0.001
60-119	2.68 (1.81-3.97)	<0.001	3.22 (2.12-4.87)	<0.001
120-239	2.42 (1.58-3.70)	<0.001	2.72 (1.75-4.24)	<0.001
240-360	1.77 (0.63-4.95)	0.280	1.95 (0.67-5.66)	0.219
Year of RC diagnosis	3.21 (2.42-4.28)	<0.001	2.84 (2.15-3.74)	<0.001
1975-1984	2.77 (1.55-4.97)	0.001	2.67 (1.48-4.83)	0.001
1985-1994	3.69 (2.28-5.95)	<0.001	3.58 (2.21-5.79)	<0.001
1995-2004	2.75 (1.64-4.62)	<0.001	2.72 (1.62-4.59)	<0.001
≥ 2005	4.82 (1.74-13.38)	0.003	5.00 (1.80-13.92)	0.002

NOTE. Poisson regression analyses were used to calculate the radiation-attributed risk (RR) and 95% confidence intervals (CIs) of SGMs for patients with radiation therapy versus patients without radiation therapy. Adjusted RRs were stratified by age at RC diagnosis and calendar year of RC diagnosis. A determination of statistical significance of RRs was based on $P<0.05$ (two sided).

Abbreviations: RC, rectal cancer; RR, radiation-attributed risk; SGM, second gynecological malignancy.

© 2021 Guan X et al. *JAMA Network Open.*

eTable 15. Radiation-attributed Risk of Ovarian Cancer by Age at RC Diagnosis, Latency and Year of RC Diagnosis.

Characteristic	RR (95% Cl)	P	Adjusted RR (95% Cl)	P
Age at RC diagnosis, years	2.15 (1.27-3.65)	0.004	2.17 (1.26-3.74)	0.005
20-49	4.05 (0.37-44.70)	0.253	4.63 (0.40-54.17)	0.222
50-69	2.02 (1.07-3.80)	0.030	2.19 (1.13-4.21)	0.020
≥ 70	2.27 (0.78-6.65)	0.134	2.02 (0.67-6.12)	0.213
Latency between RC and SGM, months	1.82 (1.07-3.09)	0.026	2.12 (1.22-3.67)	0.008
60-119	0.70 (0.26-1.89)	0.481	0.70 (0.26-1.89)	0.481
120-239	2.26 (1.09-4.70)	0.029	2.26 (1.09-4.70)	0.029
240-360	11.47 (2.23-59.14)	0.004	11.84 (2.18-64.33)	0.004
Year of RC diagnosis	2.05 (1.19-3.52)	0.009	2.15 (1.26-3.65)	0.005
1975-1984	4.86 (1.42-16.61)	0.012	5.06 (1.46-17.55)	0.011
1985-1994	3.09 (1.36-7.00)	0.007	3.05 (1.34-6.96)	0.008
1995-2004	0.94 (0.39-2.32)	0.902	1.02 (0.41-2.52)	0.967
≥ 2005	1.72 (0.11-27.51)	0.701	1.93 (0.12-31.02)	0.643

NOTE. Poisson regression analyses were used to calculate the radiation-attributed risk (RR) and 95% confidence intervals (CIs) of SGMs for patients with radiation therapy versus patients without radiation therapy. Adjusted RRs were stratified by age at RC diagnosis and calendar year of RC diagnosis. A determination of statistical significance of RRs was based on $P<0.05$ (two sided).

Abbreviations: RC, rectal cancer; RR, radiation-attributed risk; SGM, second gynecological malignancy.

eTable 16. Radiation-attributed Risk of Cervical Cancer by Age at RC Diagnosis, Latency and Year of RC Diagnosis.

Characteristic	RR (95% Cl)	P	Adjusted RR (95% Cl)	P
Age at RC diagnosis, years	1.39 (0.48-4.04)	0.541	1.47 (0.49-4.37)	0.489
20-49	1.01 (0.09-11.18)	0.010	1.27 (0.11-14.94)	0.847
50-69	2.42 (0.65-9.01)	0.188	2.13 (0.55-8.25)	0.273
≥ 70	0.00 (0.00-0.00)	1	0.00 (0.00-0.00)	1
Latency between RC and SGM, months	1.11 (0.39-3.20)	0.844	1.18 (0.39-3.56)	0.770
60-119	1.49 (0.49-4.54)	0.690	1.55 (0.48-5.01)	0.462
120-239	0.00 (0.00-0.00)	1	0.00 (0.00-0.00)	1
240-360	0.00 (0.00-0.00)	1	0.00 (0.00-0.00)	1
Year of RC diagnosis	1.50 (0.50-4.45)	0.730	1.39 (0.48-4.04)	0.542
1975-1984	0.00 (0.00-0.00)	1	0.00 (0.00-0.00)	1
1985-1994	0.56 (0.07-4.67)	0.593	0.53 (0.06-4.42)	0.556
1995-2004	2.02 (0.13-32.37)	0.618	1.95 (0.12-31.99)	0.639
≥ 2005	0.00 (0.00-0.00)	1	0.00 (0.00-0.00)	1

NOTE. Poisson regression analyses were used to calculate the radiation-attributed risk (RR) and 95% confidence intervals (CIs) of SGMs for patients with radiation therapy versus patients without radiation therapy. Adjusted RRs were stratified by age at RC diagnosis and calendar year of RC diagnosis. A determination of statistical significance of RRs was based on P<0.05 (two sided).

Abbreviations: RC, rectal cancer; RR, radiation-attributed risk; SGM, second gynecological malignancy.

© 2021 Guan X et al. *JAMA Network Open.*

eTable 17. Radiation-attributed Risk of Other SGMs by Age at RC Diagnosis, Latency and Year of RC Diagnosis.

Characteristic	RR (95% Cl)	P	Adjusted RR (95% Cl)	P
Age at RC diagnosis, years	2.40 (1.30-4.43)	0.005	2.68 (1.42-5.06)	0.002
20-49	6.08 (0.63-58.45)	0.118	6.51 (0.65-65.48)	0.112
50-69	2.27 (1.07-4.80)	0.032	2.79 (1.28-6.08)	0.010
≥ 70	1.95 (0.50-7.53)	0.334	1.75 (0.43-7.05)	0.431
Latency between RC and SGM, months	2.05 (1.11-3.80)	0.021	2.48 (1.30-4.73)	0.006
60-119	1.10 (0.42-2.89)	0.851	1.20 (0.44-3.30)	0.724
120-239	2.78 (1.11-7.01)	0.030	3.66 (1.38-9.71)	0.009
240-360	6.88 (1.15-41.20)	0.035	7.83 (1.22-50.19)	0.030
Year of RC diagnosis	2.70 (1.42-5.11)	0.002	2.40 (1.29-4.44)	0.005
1975-1984	1.42 (0.32-6.34)	0.648	1.30 (0.29-5.87)	0.732
1985-1994	3.37 (1.09-10.45)	0.035	3.56 (1.14-11.16)	0.029
1995-2004	2.83 (0.90-8.93)	0.075	3.03 (0.95-9.66)	0.060
≥ 2005	5.16 (0.54-49.63)	0.155	2.40 (1.29-4.44)	0.005

NOTE. Poisson regression analyses were used to calculate the radiation-attributed risk (RR) and 95% confidence intervals (CIs) of SGMs for patients with radiation therapy versus patients without radiation therapy. Adjusted RRs were stratified by age at RC diagnosis and calendar year of RC diagnosis. A determination of statistical significance of RRs was based on P<0.05 (two sided).

Abbreviations: RC, rectal cancer; RR, radiation-attributed risk; SGM, second gynecological malignancy.

© 2021 Guan X et al. *JAMA Network Open.*

eTable 18. Standardized Incidence Ratio of Combined SGMs by Age at RC Diagnosis, Latency and Year of RC Diagnosis.

Characteristic	RC patients treated with NRT			RC patients treated with RT		
	Observed SGMs	Expected SGMs	SIR (95% Cl)	Observed SGMs	Expected SGMs	SIR (95% Cl)
All patients	178	222.66	0.80 (0.69-0.93) #	144	62.15	2.32 (1.95-2.73) #
Age at RC diagnosis, years						
20-49	28	25.04	1.12 (0.74-1.62)	14	10.21	1.37 (0.75-2.3)
50-69	111	136.35	0.81 (0.67-0.98) #	102	39.31	2.59 (2.12-3.15) #
≥ 70	39	61.27	0.64 (0.45-0.87) #	28	12.63	2.22 (1.47-3.21) #
Latency between RC and SGM, months						
60-119	85	95.55	0.89 (0.71-1.1)	69	30.15	2.29 (1.78-2.9) #
120-239	74	95.46	0.78 (0.61-0.97) #	62	26.15	2.37 (1.82-3.04) #
240-359	17	27.48	0.62 (0.36-0.99) #	13	5.33	2.44 (1.3-4.17) #
≥ 360	2	4.17	0.48 (0.06-1.73)	0	0.52	0 (0-7.13)
Year of RC diagnosis						
1975-1984	69	88.11	0.78 (0.61-0.99) #	21	9.92	2.12 (1.31-3.23) #
1985-1994	56	72.73	0.77 (0.58-1) #	53	21.41	2.48 (1.85-3.24) #
1995-2004	46	49.76	0.92 (0.68-1.23)	49	24.07	2.04 (1.51-2.69) #
≥ 2005	7	12.06	0.58 (0.23-1.2)	21	6.74	3.11 (1.93-4.76) #

NOTE. SIR was defined as the ratio of the observed cases of second gynecological malignancy (SGM) among rectal cancer (RC) survivors to the expected number of cases in the US general population and was stratified by age at RC diagnosis and calendar year of RC diagnosis. The statistical significance of SIRs was based on a P<0.05 (two sided); 95% confidence intervals were calculated by Poisson exact methods. The background incidence of SGMs was derived from data provided by the SEER database. # indicates P<0.05.

© 2021 Guan X et al. *JAMA Network Open.*

Abbreviations: RC, rectal cancer; RT, radiation therapy; NRT, no radiation therapy; SIR, standardized incidence ratio; SGM, second gynecological malignancy.

© 2021 Guan X et al. *JAMA Network Open.*

eTable 19. Standardized Incidence Ratio of Corpus Uteri Cancer by Age at RC Diagnosis, Latency and Year of RC Diagnosis.

Characteristic	RC patients treated with NRT			RC patients treated with RT		
	Observed CUC	Expected CUC	SIR (95% Cl)	Observed CUC	Expected CUC	SIR (95% Cl)
All patients	108	116.35	0.93 (0.76-1.12)	98	34.08	2.88 (2.33-3.5) #
Age at RC diagnosis, years						
20-49	24	14.33	1.68 (1.07-2.49) #	8	5.98	1.34 (0.58-2.64)
50-69	66	74.47	0.89 (0.69-1.13)	70	22.27	3.14 (2.45-3.97) #
≥ 70	18	27.55	0.65 (0.39-1.03)	20	5.83	3.43 (2.1-5.3) #
Latency between RC and SGM, months						
60-119	47	50.73	0.93 (0.68-1.23)	53	16.72	3.17 (2.37-4.15) #
120-239	46	49.89	0.92 (0.68-1.23)	40	14.27	2.80 (2-3.82) #
240-359	13	13.77	0.94 (0.5-1.61)	5	2.81	1.78 (0.58-4.15)
≥ 360	2	1.96	1.02 (0.12-3.69)	0	0.27	0 (0-13.5)
Year of RC diagnosis						
1975-1984	46	44.82	1.03 (0.75-1.37)	15	5.23	2.87 (1.6-4.73) #
1985-1994	32	37.52	0.85 (0.58-1.2)	35	11.37	3.08 (2.14-4.28) #
1995-2004	25	26.95	0.93 (0.6-1.37)	34	13.49	2.52 (1.75-3.52) #
≥ 2005	5	7.06	0.71 (0.23-1.65)	14	3.99	3.51 (1.92-5.89) #

NOTE. SIR was defined as the ratio of the number of observed corpus uteri cancer (CUC) cases among rectal cancer (RC) survivors to the expected number of cases in the US general population and was stratified by age at RC diagnosis and calendar year of RC diagnosis. A determination of the statistical significance of SIRs was based on a $P<0.05$ (two sided). 95% confidence intervals were calculated by Poisson exact methods. The background incidence of CUC was derived from data provided by the SEER database. # indicates $P<0.05$.

Abbreviations: RC, rectal cancer; RT, radiation therapy; NRT, no radiation therapy; SIR, standardized incidence ratio; SGM, second gynecological malignancy; CUC, corpus uteri cancer.

© 2021 Guan X et al. *JAMA Network Open.*

eTable 20. Standardized Incidence Ratio of Ovarian Cancer by Age at RC Diagnosis, Latency and Year of RC Diagnosis.

Characteristic	RC patients treated with NRT			RC patients treated with RT		
	Observed OC	Expected OC	SIR (95% Cl)	Observed OC	Expected OC	SIR (95% Cl)
All patients	35	64.99	0.54 (0.38-1.75) #	23	17.17	1.34 (0.85-2.01)
Age at RC diagnosis, years						
20-49	1	6.14	0.16 (0-0.91) #	2	2.39	0.84 (0.1-3.02)
50-69	24	38.83	0.62 (0.4-0.92) #	16	10.66	1.5 (0.86-2.44)
≥ 70	10	20.02	0.50 (0.24-0.92) #	5	4.12	1.21 (0.39-2.83)
Latency between RC and SGM, months						
60-119	17	27.34	0.62 (0.36-1) #	5	8.23	0.61 (0.2-1.42)
120-239	16	28	0.57 (0.33-0.93) #	13	7.28	1.79 (0.95-3.05)
240-359	2	8.37	0.24 (0.03-0.86) #	5	1.52	3.28 (1.07-7.66) #
≥ 360	0	1.28	0 (0-2.89)	0	0.14	0 (0-26.24)
Year of RC diagnosis						
1975-1984	7	26.68	0.26 (0.11-0.54) #	4	2.94	1.36 (0.37-3.48)
1985-1994	12	21.82	0.55 (0.28-0.96) #	11	6.29	1.75 (0.87-3.13)
1995-2004	15	13.69	1.1 (0.61-1.81)	7	6.4	1.09 (0.44-2.26)
≥ 2005	1	2.8	0.36 (0.01-1.99)	1	1.55	0.65 (0.02-3.6)

NOTE. SIR was defined as the ratio of the number of observed second gynecological malignancy (SGM) cases among rectal cancer (RC) survivors to the number of expected cases in the US general population and was stratified by age at RC diagnosis and calendar year of RC diagnosis. The statistical significance of SIRs was based on a P<0.05 (two sided); 95% confidence intervals were calculated by Poisson exact methods. The background incidence of SGMs was derived from data provided by the SEER database. # indicates P<0.05.

Abbreviations: RC, rectal cancer; RT, radiation therapy; NRT, no radiation therapy; SIR, standardized incidence ratio; SGM, second gynecological malignancy; OC, ovarian cancer.

© 2021 Guan X et al. *JAMA Network Open.*

eTable 21. Standardized Incidence Ratio of Cervical Cancer by Age at RC Diagnosis, Latency and Year of RC Diagnosis.

Characteristic	RC patients treated with NRT			RC patients treated with RT		
	Observed CC	Expected CC	SIR (95% Cl)	Observed CC	Expected CC	SIR (95% Cl)
All patients	11	16.85	0.65 (0.33-1.17)	5	4.45	1.12 (0.37-2.62)
Age at RC diagnosis, years						
20-49	2	2.74	0.73 (0.09-2.64)	1	1.11	0.9 (0.02-5.03)
50-69	5	9.71	0.51 (0.17-1.2)	4	2.59	1.55 (0.42-3.96)
≥ 70	4	4.4	0.91 (0.25-2.33)	0	0.75	0 (0-4.93)
Latency between RC and SGM, months						
60-119	8	8.11	0.99 (0.43-1.94)	5	2.38	2.1 (0.68-4.91)
120-239	3	6.91	0.43 (0.09-1.27)	0	1.74	0 (0-2.12)
240-359	0	1.62	0 (0-2.28)	0	0.3	0 (0-12.34)
≥ 360	0	0.21	0 (0-17.75)	0	0.03	0 (0-136.54)
Year of RC diagnosis						
1975-1984	4	7.48	0.53 (0.15-1.37)	0	0.84	0 (0-4.39)
1985-1994	6	5.29	1.13 (0.42-2.47)	1	1.55	0.64 (0.02-3.58)
1995-2004	1	3.26	0.31 (0.01-1.71)	1	1.59	0.63 (0.02-3.51)
≥ 2005	0	0.82	0 (0-4.51)	3	0.46	6.47 (1.33-18.92) #

NOTE. SIR was defined as the ratio of the number of observed second gynecological malignancy (SGM) cases among rectal cancer (RC) survivors to the expected number of cases in the US general population and was stratified by age at RC diagnosis and calendar year of RC diagnosis. The statistical significance of SIRs was based on a P<0.05 (two sided); 95% confidence intervals were calculated by Poisson exact methods. The background incidence of SGMs was derived from data provided by the SEER database. # indicates P<0.05.

Abbreviations: RC, rectal cancer; RT, radiation therapy; NRT, no radiation therapy; SIR, standardized incidence ratio; SGM, second gynecological malignancy; CC, cervical cancer.

© 2021 Guan X et al. *JAMA Network Open.*

eTable 22. Standardized Incidence Ratio of Other SGMs by Age at RC Diagnosis, Latency and Year of RC Diagnosis.

Characteristic	RC patients treated with NRT			RC patients treated with RT		
	Observed SGMs	Expected SGMs	SIR (95% Cl)	Observed SGMs	Expected SGMs	SIR (95% Cl)
All patients	24	24.46	0.98 (0.63-1.46)	18	6.45	2.79 (1.65-4.41) #
Age at RC diagnosis, years						
20-49	1	1.83	0.55 (0.01-3.04)	3	0.73	4.13 (0.85-12.08)
50-69	16	13.33	1.2 (0.69-1.95)	12	3.79	3.16 (1.63-5.53) #
≥ 70	7	9.3	0.75 (0.3-1.55)	3	1.93	1.55 (0.32-4.54)
Latency between RC and SGM, months						
60-119	13	9.37	1.39 (0.74-2.37)	6	2.82	2.13 (0.78-4.64)
120-239	9	10.66	0.84 (0.39-1.6)	9	2.85	3.15 (1.44-5.99) #
240-359	2	3.72	0.54 (0.07-1.94)	3	0.7	4.27 (0.88-12.47)
≥ 360						
Year of RC diagnosis						
1975-1984	12	9.13	1.31 (0.68-2.3)	2	0.91	2.2 (0.27-7.94)
1985-1994	6	8.1	0.74 (0.27-1.61)	6	2.19	2.74 (1-5.96) #
1995-2004	5	5.85	0.86 (0.28-2)	7	2.6	2.69 (1.08-5.54) #
≥ 2005	1	1.38	0.72 (0.02-4.03)	3	0.75	4.02 (0.83-11.75)

NOTE. SIR was defined as the ratio of the number of observed second gynecological malignancy (SGM) cases among rectal cancer (RC) survivors to the expected number of cases in the US general population and was stratified by age at RC diagnosis and calendar year of RC diagnosis. A determination of the statistical significance of SIRs was based on a P<0.05 (two sided); 95% confidence intervals were calculated by Poisson exact methods. The background incidence of SGMs was derived from data provided by the SEER database. # indicates P<0.05.

Abbreviations: RC, rectal cancer; RT, radiation therapy; NRT, no radiation therapy; SIR, standardized incidence ratio; SGM, second gynecological malignancy.

© 2021 Guan X et al. *JAMA Network Open.*

eTable 23. Patients Characteristics of Corpus Uteri Cancer and Matched Only Primary Corpus Uteri Cancer.

Characteristic	CUC (NRT) (n=106)	OPCUC matched with CUC (NRT) (n=530)	P	CUC (RT) (n=98)	OPCUC matched with CUC (RT) (n=490)	P
Age at RC diagnosis, No. (%), years			0.997			0.938
20-49	7 (6.6)	34 (6.4)		1 (1.1)	7 (1.4)	
50-69	36 (34)	180 (34)		37 (37.8)	180 (36.8)	
≥ 70	63 (59.4)	316 (59.6)		60 (61.1)	303 (61.8)	
Year of RC diagnosis, No. (%)			0.316			0.944
1975-1984	3 (2.8)	34 (6.4)		1 (1.1)	8 (1.6)	
1985-1994	27 (25.5)	128 (24.2)		10 (10.2)	53 (10.8)	
1995-2004	33 (31.1)	133 (25.1)		31 (31.6)	144 (29.4)	
≥ 2005	43 (40.6)	235 (44.3)		56 (57.1)	285 (58.2)	
Race, No. (%)			0.613			0.647
White	91 (85.8)	454 (85.7)		82 (83.7)	401 (81.8)	
Black	4 (3.8)	24 (4.5)		5 (5.1)	38 (7.8)	
Other	11 (10.4)	52 (9.8)		11 (11.2)	51 (10.4)	

© 2021 Guan X et al. *JAMA Network Open.*

Tumor grade, No. (%)			0.906			0.966
Grade I/II	49 (46.2)	256 (48.3)		13 (13.3)	70 (14.3)	
Grade III/IV	36 (34)	169 (31.9)		54 (55.1)	267 (54.5)	
Unknown	21 (19.8)	105 (19.8)		31 (30.7)	153 (31.2)	
Tumor stage, No. (%)			0.973			0.951
Localized	72 (67.9)	364 (68.7)		37 (37.8)	189 (38.6)	
Regional	20 (18.9)	102 (19.2)		29 (29.6)	135 (27.6)	
Distant	9 (8.5)	38 (7.2)		18 (18.4)	100 (20.3)	
Unstaged	5 (4.7)	26 (4.9)		14 (14.2)	66 (13.5)	
Tumor histology, No. (%)			0.944			0.544
Adenocarcinoma	79 (74.5)	399 (75.3)		44 (44.9)	220 (44.9)	
Mucous tumor	8 (7.5)	46 (8.7)		20 (20.4)	93 (19)	
Epithelial Neoplasm	9 (8.5)	38 (7.2)		3 (3.1)	6 (1.2)	
Other	10 (9.5)	47 (8.9)		31 (31.6)	171 (34.9)	
Surgery, No. (%)			0.805			1
No	17 (16)	80 (15.1)		19 (19.4)	95 (19.4)	
Yes	89 (84)	450 (84.9)		79 (80.6)	395 (80.6)	

© 2021 Guan X et al. *JAMA Network Open.*

Chemotherapy, No. (%)			0.810			0.969
No	88 (83)	445 (84)		65 (66.3)	324 (66.1)	
Yes	18 (17)	85 (16)		33 (33.7)	166 (33.9)	
Radiation, No. (%)			0.691			1
No	83 (78.3)	424 (80)		88 (89.8)	440 (89.8)	
Yes	23 (21.7)	106 (20)		10 (10.2)	50 (10.2)	

NOTE. Rectal cancer (RC) patients who developed corpus uteri cancer (CUC) were matched to patients with only primary CUC (OPCUC), with a PSM ratio of 1:5 for CUC versus OPCUC patients. The variables matched for PSM included age at gynecological malignancy (GM) diagnosis, year of GM diagnosis, race, stage of GM and type of GM treatment.

Abbreviations: RC, rectal cancer; RT, radiation therapy; NRT, no radiation therapy; GM, gynecological malignancy; CUC, corpus uteri cancer; OPCUC, only primary corpus uteri cancer.

© 2021 Guan X et al. *JAMA Network Open.*

eTable 24. Patients Characteristics of Ovarian Cancer and Matched Only Primary Ovarian Cancer.

Characteristic	OC (NRT) (n=35)	OPOC matched with OC (NRT) (n=175)	P	OC (RT) (n=23)	OPOC matched with OC (RT) (n=115)	P
Age at RC diagnosis, No. (%), years			0.893			0.495
20-49	0 (0)	0 (0)		0 (0)	0 (0)	
50-69	11 (31.4)	53 (30.3)		5 (21.7)	33 (28.7)	
≥ 70	24 (68.6)	122 (69.7)		18 (78.3)	82 (71.3)	
Year of RC diagnosis, No. (%)			0.841			0.486
1975-1984	0 (0)	0 (0)		0 (0)	0 (0)	
1985-1994	9 (25.7)	39 (22.3)		3 (13)	7 (6.1)	
1995-2004	10 (28.6)	58 (33.1)		5 (21.7)	30 (26.1)	
≥ 2005	16 (45.7)	78 (44.6)		15 (15.5)	78 (67.8)	
Race, No. (%)			0.981			0.818
White	26 (74.3)	131 (74.9)		22 (95.7)	111 (96.5)	

© 2021 Guan X et al. *JAMA Network Open.*

Black	5 (14.3)	23 (13.1)		0 (0)	1 (0.9)	
Other	4 (11.4)	21 (12)		1 (4.3)	3 (2.6)	
Tumor grade, No. (%)			0.992			0.808
Grade I/II	9 (25.7)	46 (26.2)		2 (8.7)	6 (5.2)	
Grade III/IV	14 (40)	68 (38.9)		8 (34.8)	41 (35.7)	
Unknown	12 (34.3)	61 (34.9)		13 (56.5)	68 (59.1)	
Tumor stage, No. (%)			0.607			0.769
Localized	7 (20)	22 (12.6)		3 (13)	11 (9.6)	
Regional	2 (5.7)	18 (10.3)		2 (8.7)	15 (13)	
Distant	25 (71.4)	130 (74.2)		18 (78.3)	89 (77.4)	
Unstaged	1 (2.9)	5 (2.9)		0 (0)	0 (0)	
Tumor histology, No. (%)			0.845			0.9147
Adenocarcinoma	11 (31.4)	61 (34.9)		11 (47.8)	55 (47.8)	
Mucous tumor	16 (45.7)	80 (45.7)		8 (34.8)	41 (35.7)	
Epithelial Neoplasm	2 (5.7)	13 (7.4)		2 (8.7)	13 (11.3)	

© 2021 Guan X et al. *JAMA Network Open.*

Other	6 (17.2)	21 (12)		2 (8.7)	6 (5.2)	
Surgery, No. (%)			0.834			0.817
No	7 (20)	38 (21.6)		9 (39.1)	48 (41.7)	
Yes	28 (80)	138 (78.4)		14 (60.9)	67 (67.5)	
Chemotherapy, No. (%)			0.804			0.697
No	20 (57.1)	96 (54.9)		10 (43.5)	45 (39.1)	
Yes	15 (42.9)	79 (45.1)		13 (56.5)	70 (60.9)	
Radiation, No. (%)			0.525			1
No	35 (100)	173 (98.9)		0 (0)	0 (0)	
Yes	0 (0)	2 (1.1)		23 (100)	115 (100)	

NOTE. Rectal cancer (RC) patients who developed ovarian cancer (OC) were matched with patients with only primary OC (OPOC) at a PSM ratio of 1:5 for OC patients versus OPOC patients. The matched variables for PSM included age at gynecological malignancy (GM) diagnosis, year of GM diagnosis, race, stage of GM and treatment type of GM.

Abbreviations: RC, rectal cancer; RT, radiation therapy; NRT, no radiation therapy; GM, gynecological malignancy; OC, ovarian cancer; OPOC, only primary ovarian cancer.

© 2021 Guan X et al. *JAMA Network Open.*

eTable 25. Patients Characteristics of Cervical Cancer and Matched Only Primary Cervical Cancer.

Characteristic	CC (NRT) (n=11)	OPCC matched with CC (NRT) (n=55)	P	CC (RT) (n=5)	OPCC matched with CC (RT) (n=25)	P
Age at RC diagnosis, No. (%), years			0.136			0.249
20-49	2 (18.1)	1 (1.8)		0 (0)	3 (12)	
50-69	4 (36.4)	23 (41.8)		4 (80)	10 (40)	
≥ 70	5 (45.5)	31 (56.4)		1 (20)	12 (48)	
Year of RC diagnosis, No. (%)			0.596			0.458
1975-1984	1 (9.1)	11 (20)		0 (0)	0 (0)	
1985-1994	4 (36.3)	15 (27.3)		1 (20)	3 (12)	
1995-2004	5 (45.5)	18 (32.7)		0 (0)	6 (24)	
≥ 2005	1 (9.1)	11 (20)		4 (80)	16 (64)	
Race, No. (%)			0.427			1
White	7 (63.6)	41 (74.5)		5 (100)	25 (100)	
Black	2 (18.2)	11 (20)		0 (0)	0 (0)	
Other	2 (18.2)	3 (5.5)		0 (0)	0 (0)	
Tumor grade, No. (%)			0.863			0.977

© 2021 Guan X et al. *JAMA Network Open.*

Grade I/II	3 (27.3)	17 (30.9)		2 (40)	9 (36)	
Grade III/IV	3 (27.3)	11 (20)		1 (20)	6 (24)	
Unknown	5 (45.4)	27 (49.1)		2 (40)	10 (40)	
Tumor stage, No. (%)			0.143			0.373
Localized	5 (45.5)	32 (58.2)		1 (20)	1 (4)	
Regional	5 (45.5)	23 (41.8)		2 (40)	6 (24)	
Distant	0 (0)	0 (0)		1 (20)	14 (56)	
Unstaged	1 (9)	0 (0)		1 (20)	4 (16)	
Tumor histology, No. (%)			0.566			0.944
Adenocarcinoma	5 (45.5)	17 (30.9)		2 (40)	12 (48)	
Mucous tumor	0 (0)	1 (1.8)		0 (0)	0 (0)	
Epithelial Neoplasm	0 (0)	0 (0)		1 (20)	4 (16)	
Other	6 (54.5)	37 (67.3)		2 (40)	9 (36)	
Surgery, No. (%)			0.912			0.593
No	5 (45.5)	24 (43.6)		3 (60)	18 (72)	
Yes	6 (54.5)	31 (56.4)		2 (40)	7 (28)	
Chemotherapy, No. (%)			0.890			0.847

© 2021 Guan X et al. *JAMA Network Open.*

No	9 (81.8)	44 (80)		1 (20)	6 (24)	
Yes	2 (18.2)	11 (20)		4 (80)	19 (76)	
Radiation, No. (%)			0.630			0.593
No	9 (81.8)	48 (87.3)		4 (80)	17 (68)	
Yes	2 (18.2)	7 (12.7)		1 (20)	8 (32)	

NOTE. Rectal cancer (RC) patients who developed cervical cancer (CC) were matched to patients with only primary CC (OPCC) at a PSM of 1:5 for CC versus OPCC. The variables matched for PSM included age at gynecological malignancy (GM) diagnosis, year of GM diagnosis, race, stage of GM and type of GM treatment.

Abbreviations: RC, rectal cancer; RT, radiation therapy; NRT, no radiation therapy; GM, gynecological malignancy; CC, cervical cancer; OPCC, only primary cervical cancer.

© 2021 Guan X et al. *JAMA Network Open.*

eTable 26. Patients Characteristics of Other SGMs and Matched Other Only Primary SGMs.

Characteristic	Other SGM patients (NRT) (n=24)	OPSGM patients matched with other SGM patients (NRT) (n=120)	P	Other SGM patients (RT) (n=18)	OPSGM patients matched with other SGM patients (RT) (n=90)	P
Age at RC diagnosis, No. (%), years			0.593			0.655
20-49	0 (0)	5 (4.2)		1 (5.6)	2 (2.2)	
50-69	5 (20.8)	23 (19.1)		5 (27.8)	21 (23.3)	
≥ 70	19 (79.2)	92 (76.7)		12 (66.6)	67 (74.5)	
Year of RC diagnosis, No. (%)			0.426			0.660
1975-1984	2 (8.3)	13 (10.9)		0 (0)	0 (0)	
1985-1994	8 (33.3)	24 (20)		0 (0)	1 (1.1)	
1995-2004	7 (29.2)	52 (43.3)		5 (27.8)	28 (31.1)	
≥ 2005	7 (29.2)	31 (25.8)		13 (72.2)	61 (67.8)	
Race, No. (%)			0.958			0.496
White	22 (91.6)	112 (93.4)		16 (88.9)	85 (94.4)	
Black	1 (4.2)	4 (3.3)		2 (11.1)	4 (4.4)	

© 2021 Guan X et al. *JAMA Network Open.*

Other	1 (4.2)	4 (3.3)		0 (0)	1 (1.2)	
Tumor grade, No. (%)			0.935			0.555
Grade I/II	7 (29.2)	31 (25.8)		6 (33.3)	24 (26.7)	
Grade III/IV	7 (29.2)	35 (29.2)		3 (16.7)	26 (28.9)	
Unknown	10 (41.6)	54 (45)		9 (50)	40 (44.4)	
Tumor stage, No. (%)			0.845			0.827
Localized	9 (37.5)	49 (40.8)		6 (33.3)	37 (41.1)	
Regional	10 (41.7)	44 (36.7)		2 (11.1)	9 (10)	
Distant	2 (8.3)	16 (13.3)		0 (0)	0 (0)	
Unstaged	3 (12.5)	11 (9.2)		10 (55.6)	44 (48.9)	
Tumor histology, %			0.617			0.680
Adenocarcinoma	7 (29.2)	26 (21.7)		3 (16.7)	13 (14.5)	
Mucous tumor	0 (0)	5 (4.2)		0 (0)	3 (3.3)	
Epithelial Neoplasm	2 (8.3)	7 (5.8)		0 (0)	4 (4.4)	
Other	15 (62.5)	82 (68.3)		15 (83.3)	70 (77.8)	
Surgery, No. (%)			0.754			1
No	9 (37.5)	41 (34.2)		9 (50)	44 (48.9)	

© 2021 Guan X et al. *JAMA Network Open.*

Yes	15 (62.5)	79 (65.8)		9 (50)	46 (51.1)	
Chemotherapy, No. (%)			0.761			0.658
No	15 (62.5)	71 (59.2)		14 (77.8)	74 (82.2)	
Yes	9 (37.5)	49 (40.8)		4 (22.2)	16 (17.8)	
Radiation, No. (%)			0.679			0.640
No	21 (87.5)	101 (84.2)		16 (88.9)	83 (92.2)	
Yes	3 (12.5)	19 (15.8)		2 (11.1)	7 (7.8)	

NOTE. Rectal cancer (RC) patients who developed other SGMs were matched with patients with only primary SGMs (OPSGMs) at a PSM ratio of 1:5 for other SGMs versus OPSGMs. The matched variables for PSM included age at gynecological malignancy (GM) diagnosis, year of GM diagnosis, race, stage of GM and type of GM treatment.

Abbreviations: RC, rectal cancer; RT, radiation therapy; NRT, no radiation therapy; GM, gynecological malignancy; SGM, second gynecological malignancy; OPSGM, only primary gynecological malignancy.

© 2021 Guan X et al. *JAMA Network Open.*

eTable 27. Patients Characteristics of Corpus Uteri Cancer Before and After PSM Matching.

Characteristic	Before PSM			After PSM		
	CUC (NRT) (n=106)	CUC (RT) (n=98)	P	CUC (NRT) (n=81)	CUC (RT) (n=81)	P
Age at RC diagnosis, No. (%), years			0.118			0.774
20-49	7 (6.6)	1 (1.1)		6 (7.4)	1 (1.2)	
50-69	36 (34)	37 (37.8)		24 (29.6)	32 (39.5)	
≥ 70	63 (59.4)	60 (61.1)		51 (63)	48 (59.3)	
Year of RC diagnosis, No. (%)			0.016			0.125
1975-1984	3 (2.8)	1 (1.1)		3 (3.7)	1 (1.2)	
1985-1994	27 (25.5)	10 (10.2)		16 (19.8)	10 (12.3)	
1995-2004	33 (31.1)	31 (31.6)		26 (32.1)	28 (34.6)	
≥ 2005	43 (40.6)	56 (57.1)		36 (44.4)	42 (51.9)	
Race, No. (%)			0.875			0.97
White	91 (85.8)	82 (83.7)		68 (84)	67 (82.7)	
Black	4 (3.8)	5 (5.1)		4 (4.9)	4 (4.9)	
Other	11 (10.4)	11 (11.2)		9 (11.1)	10 (12.4)	
Tumor stage, No. (%)			<0.001			0.076

© 2021 Guan X et al. *JAMA Network Open.*

Localized	72 (67.9)	37 (37.8)		52 (64.2)	36 (44.4)	
Regional	20 (18.9)	29 (29.6)		16 (19.8)	23 (28.4)	
Distant	9 (8.5)	18 (18.4)		8 (9.9)	11 (13.6)	
Unstaged	5 (4.7)	14 (14.2)		5 (6.1)	11 (13.6)	
Surgery, No. (%)			0.531			1
No	17 (16)	19 (19.4)		14(17.3)	14 (17.3)	
Yes	89 (84)	79 (80.6)		67(82.7)	67 (82.7)	
Chemotherapy, No. (%)			0.005			0.19
No	88 (83)	65 (66.3)		66 (81.5)	59 (72.8)	
Yes	18 (17)	33 (33.7)		15 (18.5)	22 (27.2)	
Radiation, No. (%)			0.026			0.14
No	83 (78.3)	88 (89.8)		64 (79)	71 (87.7)	
Yes	23 (21.7)	10 (10.2)		17 (21)	10 (12.3)	

NOTE. Rectal cancer (RC) patients who developed corpus uteri cancer (CUC) after radiation therapy (RT) were matched to patients after no RT(NRT), with a PSM ratio of 1:1. The variables matched for PSM included age at gynecological malignancy (GM) diagnosis, year of GM diagnosis, race, stage of GM and type of GM treatment.

Abbreviations: RC, rectal cancer; RT, radiation therapy; NRT, no radiation therapy; CUC, corpus uteri cancer.

© 2021 Guan X et al. *JAMA Network Open.*

eTable 28. Studies of The Risk of Developing SGMs After Radiation Therapy Among RC patients.

Title	Journal	Study (year)	Study period	Registry	Latency period	Observation population	Total no. of patients	Study design	No. of patients in study group	No. of patients in control group	Statistical method	Median follow-up time	Types of SPMs	No. of SPMs	Types of SGMs	No. of SGM	Risk of SGMs
Occurrence of Second Cancers in Patients Treated with Radiotherapy for Rectal Cancer	J Clin Oncol	2005	1980-1990	Uppsala Trial and Swedish Rectal Cancer Trial	6 months	Rectal cancer (Stage II/III) treated with surgery	1,599	RT (pre/post) vs NRT	Unknown	Unknown	Relative risks (RRs) were calculated with 95% CIs	14 years	All types of SPMs	122	Combine CUC and CC as one group	8	No risk increased.
No Increased Risk of Second Cancer After Radiotherapy in Patients Treated for Rectal or Endometrial Cancer in the Randomized TME, PORTEC-1, and PORTEC-2 Trials	J Clin Oncol	2015	1990-2006	Three trials (TME, PORTEC-1, and PORTEC-2 trial)	Unknown	Endometrial carcinoma and rectal cancer	2,554	EBRT vs NRT	1,008	1,546	Competing-risk model and SIR	13 years	All types of SPMs	759	Each type of SGMs (CUC, OC)	10	No increased risk.
Incidence of Second Tumors after Treatment with or without Radiation for Rectal Cancer	Ann Oncol	2016	1989-2007	Netherlands population-based cancer registry	Unknown	Rectal cancer treated with surgery	29,027	RT vs NRT	15,467	13,560	Fine and Grays competing risk model and SIR	8.1 years	Pelvic tumors	4,398	Combine CUC, CC, OC, VC and other SGMs as one group	202	Increased risk from RT.
Secondary Malignancies After Rectal Cancer Resection with And Without Radiation Therapy: A Propensity-Adjusted, Population-Based SEER Analysis	Radiother Oncol	2017	1973-2012	SEER	1 year	Rectal cancer	77,484	RT vs NRT	34,114	43,370	Competing-risk model	5.8 years	All types of SPMs	7,951	Each type of SGMs (CUC, OC, CC, VC)	374	Increased risk for CUC.
A Population-Based Analysis of Second Primary Cancers After Irradiation for Rectal Cancer	Am J Clin Oncol	2007	1973-1996	SEER	5 years	Rectal cancer (Localized stage)	20,910	RT vs Non-RT	5,641	15,269	Cox proportional hazards regression	>100 months	All types of SPMs	3,024	Combine CUC and CC as one group	99	Increased risk for cancers of CUC & CC.

© 2021 Guan X et al. *JAMA Network Open.*

Second Primary Malignancy Risk After Radiotherapy in Rectal Cancer Survivors	World J Gastroenterol	2018	1996-2011	Taiwan's National Health Insurance Research Database	1 year	Rectal cancer treated with surgery	28,220	RT vs NRT	6,451	21,769	COX	5.2 years	All types of SPMs	1,270	Each type of SGMs (CC, CUC, OC)	43	Increased risk for CUC from pre-RT;<br><br>No increased risk for OC and CC.
Risk of Second Primary Cancer in Patients Treated with Radiotherapy for Rectal Cancer	Brit J Surg	2015	1980-2006	6 randomized RT rectal cancer trials conducted in SCRCR	6 months	Rectal cancer treated with surgery	13,457	RT vs NRT	7,024	6,433	Cox regression models	5.9 years in RT; 5.1 years in NRT	All types of SPMs	1,390	Each type of SGMs (CUC&CC, OC, VC)	53	No increased risk.
Multiple Primary Tumors Following Stage II and III Rectal Cancer in Patients Receiving Radiotherapy, 1998–2010	J Cancer Res Clin Oncol	2014	1992-2010	SEER	2 months	Rectal cancer (Stage II/III) treated with surgery	29,230	RT (pre) vs RT (post) vs NRT	Unknown	Unknown	Cox proportional hazards regression and SIR	Unknown	All types of SPMs	2,429	-	-	-
**The present study**	**-**	**2020**	**1973-2015**	**SEER**	**5 years**	**Rectal cancer treated with surgery**	**20,142**	**RT vs NRT**	**5,310**	**14,832**	**Fine and Grays competing risk regression and Poisson regression and SIR**	**120 months (RT);<br><br>154 months (NRT)**	**SGMs**	**320**	**Combine SGMs as one group;<br><br>Each type of SGMs (CUC, OC, CC, Other SGMs)**	**320**	**Increased risk for SGMs combined, CUC and OC.**

Abbreviations: RC, rectal cancer; RT, radiation therapy; NRT, no radiation therapy; SPM, second primary malignancy; SGM, second gynecological malignancy; CC, cervical cancer; CUC, corpus uteri cancer; OC, ovarian cancer; VC, vaginal cancer; VUC, vulvar cancer; SEER, Surveillance, Epidemiology and End Results Program cancer; SCRCR, Sweden and the Swedish ColoRectal Cancer Registry; SIR, standardized incidence ratio.

© 2021 Guan X et al. *JAMA Network Open.*
